# Optomechanical Pumping of Collective Molecular Vibrations in Plasmonic Nanocavities

**DOI:** 10.1021/acsnano.4c16535

**Published:** 2025-03-14

**Authors:** Lukas A. Jakob, Adrián Juan-Delgado, Niclas S. Mueller, Shu Hu, Rakesh Arul, Roberto A. Boto, Ruben Esteban, Javier Aizpurua, Jeremy J. Baumberg

**Affiliations:** † Nanophotonics Centre, Cavendish Laboratory, University of Cambridge, Cambridge CB3 0US, U.K.; ‡ Centro de Física de Materiales (CFM-MPC), CSIC-UPV/EHU, Paseo Manuel de Lardizabal 5 Gipuzkoa, Donostia-San Sebastián 20018, Spain; § Donostia International Physics Center (DIPC), Paseo Manuel de Lardizabal 4 Gipuzkoa, Donostia-San Sebastián 20018, Spain; ∥ Ikerbasque, Basque Foundation for Science, María Díaz de Haro 3, Bilbao 48009, Spain; ⊥ Department of Electricity and Electronics, FCT-ZTF, University of the Basque Country (UPV/EHU), Leioa 48940, Spain

**Keywords:** surface-enhanced Raman scattering, molecular optomechanics, vibrational pumping, collective vibration, NPoM

## Abstract

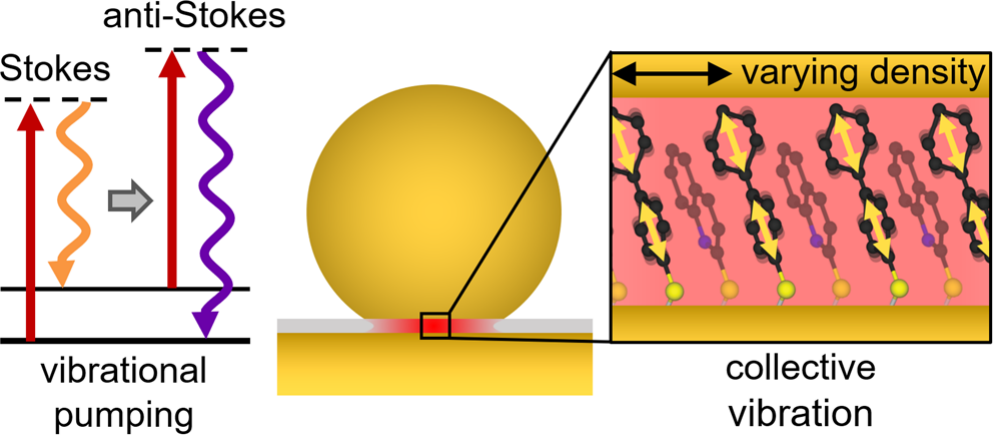

In surface-enhanced Raman scattering (SERS), vibrations of molecules couple with optical modes of a plasmonic nanocavity via a molecular optomechanical interaction. This molecule-plasmon coupling gives rise to optomechanical effects such as vibrational pumping–the excitation of molecular vibrations due to Stokes scattering. Here, we investigate the influence of vibrational pumping and collective effects on biphenyl-4-thiol (BPT) molecules in nanoparticle-on-mirror nanocavities, both experimentally by pulsed SERS spectroscopy and theoretically with optomechanical modeling. From the anti-Stokes to Stokes ratio of hundreds of individual nanostructures, we provide clear experimental evidence of vibrational pumping in high-wavenumber vibrational modes at room temperature and investigate the emergence of collective vibrational effects experimentally by varying the spacing and number of BPT molecules in the nanocavity. This is achieved by preparing mixed monolayers of different molecular species with distinct vibrational spectra. We show a 3-fold reduction of the vibrational pumping rate in experiments by tuning the collective coupling through the intermolecular spacing. Including the full plasmonic multimode response as well as collective molecular vibrations in the optomechanical theory leads to good agreement with experiments. The optomechanical control of molecular vibrations may thus enable bond-selective plasmonic chemistry, collective parametric instabilities, and phonon lasing.

## Introduction

Molecular vibrations are of increasing importance in many fields such as molecular electronics^1,2^ and bond-selective chemistry.^3,4^ Exciting the vibrations of molecules with optical techniques allows for directly influencing the rate and selectivity of chemical reactions.^4−7^ An important tool to study molecular vibrations at metal interfaces is surface-enhanced Raman scattering (SERS).^8,9^ Using the plasmonic field enhancement of SERS substrates, the measured Raman signal is increased by up to 10 orders of magnitude enabling single-molecule detection.^10−12^

**Figure 1:**
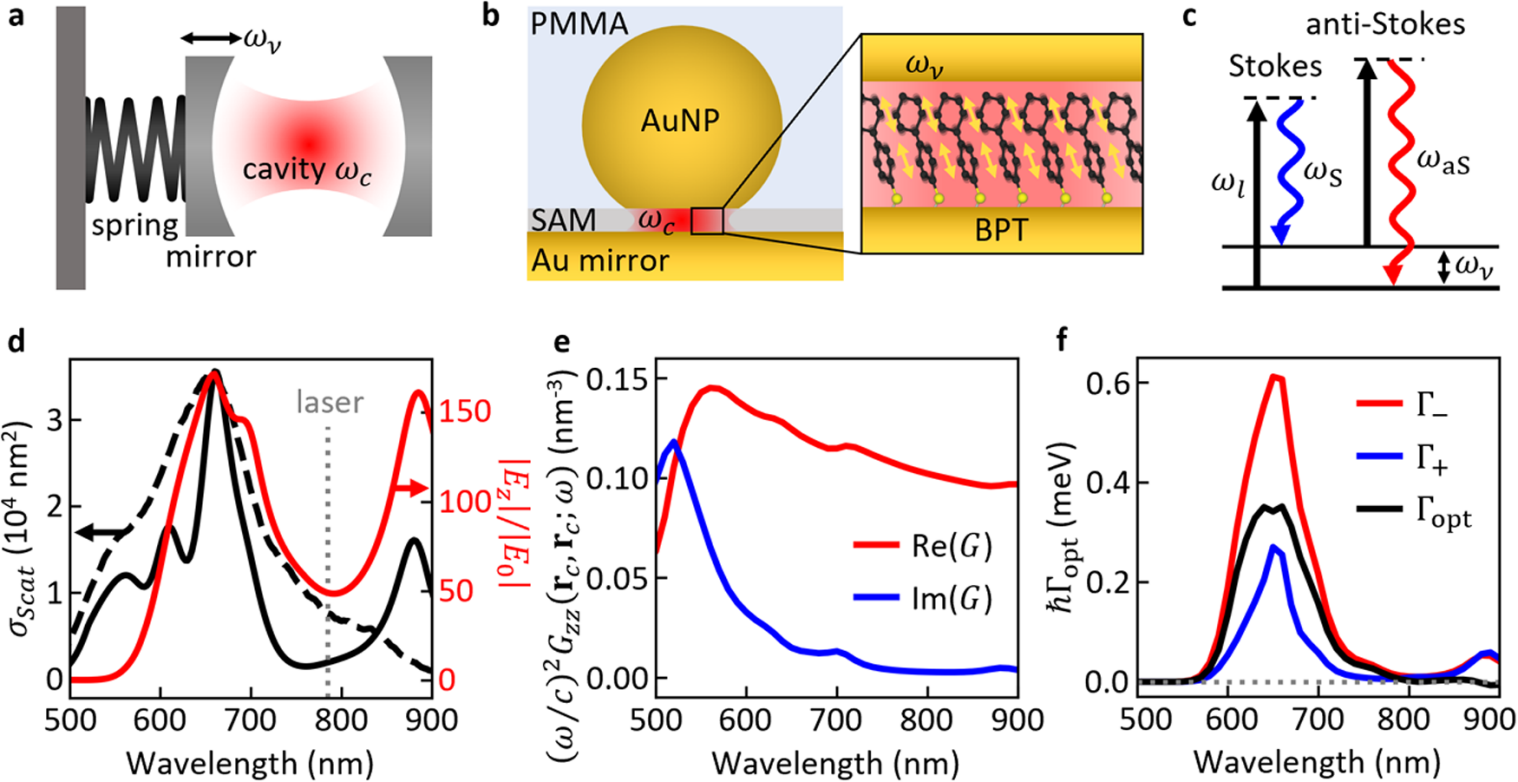
Molecular optomechanics in plasmonic nanocavities. (a) Schematic of a macroscopic optomechanical system of a cavity with one mirror attached to a spring. The optical cavity resonance at ω_c_ and the mechanical mode at ω_ν_ are coupled through the optomechanical interaction. (b) Nanoparticle-on-mirror (NPoM) plasmonic cavity confining light to a monolayer of biphenyl-4-thiol (BPT) molecules supporting a vibrational mode of frequency ω_ν_. (c) Inelastic Stokes (ω_S_) and anti-Stokes (ω_aS_) Raman scattering of a laser (ω_l_) by a vibrational mode at ω_ν_. (d) Simulated (solid black) and experimental (dashed black, normalized to simulation) scattering cross-section of the NPoM nanocavity and simulated field enhancement (red) in the center of the molecular patch **r**_c_ (introduced in the main text). (e) Simulated self-interaction Green’s function (red: real part, blue: imaginary part) of the NPoM evaluated in the center of the molecular patch, used for optomechanical simulations. (f) Optomechanical pumping (Γ_+_) and damping (Γ_–_) rates vs excitation laser wavelength for a vibrational mode at ω_ν_ = 1586 cm^–1^ (laser intensity 2 × 10^7^ μW μm^–2^). Parameters of simulations in (d–f) are given in the main text.

It has been shown that SERS can be modeled in the theoretical framework of cavity optomechanics, in this case termed “molecular optomechanics”.^13−15^ In cavity optomechanics, a mechanical vibration interacts with an optical cavity mode via optomechanical coupling giving rise to new effects such as dynamical backaction, vibrational amplification, or cooling (Figure 1a).^16^ In contrast to other optomechanical systems, the mechanical modes in SERS are provided by molecular vibrations, some of which exhibit vibrational energies ℏω_ν_ ≫ *k*_B_*T* at room temperature, and thus do not require cryogenic cooling to bring the system into the mechanical ground state. When a layer of molecules is placed in a plasmonic nanocavity (Figure 1b), the photons in the cavity interact with the molecular vibrations via surface-enhanced Stokes and anti-Stokes Raman scattering (Figure 1c). As a result, Stokes scattering excites the vibrational mode while anti-Stokes scattering depopulates it.

To fully describe this molecular optomechanical system, it is necessary to account for the complexity of plasmonic multimode cavities^17,18^ as well as collective molecular vibrations of molecules tightly packed in the nanocavity.^19−21^ Experimental evidence of molecular optomechanical effects include single-molecule vibrational pumping at low temperature,^22^ nonlinear vibrational instabilities under pulsed laser illumination,^23,24^ and a giant optomechanical spring shift supported by collective molecular vibrations.^21^ Further, molecular optomechanical systems have been utilized for the detection of mid-infrared photons by frequency upconversion to visible light.^25−27^

In the molecular optomechanical system, surface-enhanced Stokes scattering can strongly pump the population of a Raman-active vibration, a mechanism termed “vibrational pumping”.^28^ To induce a phonon population which is significant compared to the thermal population at room temperature, it is necessary to increase Stokes scattering rates using plasmonic field enhancement. Early evidence of vibrational pumping was based on the quadratic laser power dependence of the anti-Stokes signal from electronically resonant rhodamine 6G adsorbed onto metal colloids.^29^ The existence of vibrational pumping was further evidenced by temperature-dependent experiments,^30,31^ which could not be explained by plasmon resonance effects inducing anomalous anti-Stokes/Stokes ratios^32,33^ or laser-induced heating.^34^ However, in addition to the optomechanical interaction, the mechanism of vibrational pumping has also been discussed in the literature in the context of fluorescence pumping, impulsive electron-vibration excitation, and hot electrons.^28,35−37^ Thus, the comparison of experiments with the prediction of the molecular optomechanics formalism allows us to test the occurrence of vibrational pumping^15,38,39^ and discover additional effects under high-intensity excitation.^21,23,24^ Notably, this allows one to investigate whether molecules in the nanocavity can be treated independently, as done in classical models, or if collective effects induced by plasmon-mediated vibration–vibration interactions need to be included, as stressed in recent works.^19−21^

In this article, we present evidence for vibrational pumping and collective effects in high-wavenumber vibrational modes in plasmonic nanoparticle-on-mirror cavities at room temperature under pulsed laser illumination and compare experimental results to optomechanical simulations. To fully describe the experimental results, it is necessary to account for the complex multimode resonances of the plasmonic cavity and also collective molecular vibrations, here achieved by a continuum-field model of the plasmonic resonances and the simulation of >200 coupled vibrational dipoles in the cavity, respectively. Further, we provide experimental evidence that these collective vibrational modes lower the laser intensity necessary to observe the optomechanical interaction, which is achieved by tuning the spacing and number of molecules in the nanocavity. These results cannot be explained with the classical model of Raman scattering because collective vibrational effects are typically ignored.^8^

## Results and Discussion

In this work, we use nanoparticle-on-mirror (NPoM) plasmonic cavities (Figure 1b) to probe collective molecular vibrations under extreme field enhancements.^40^ A highly ordered self-assembled monolayer (SAM) of organic molecules defines a nm-thin gap between a Au nanoparticle and a mirror. Here, we use 1,1′-biphenyl-4-thiol (BPT) molecules as the spacer since they provide intense Raman activity due to their high polarizability and form well-ordered SAMs of molecules standing upright on the gold surface.^41^ The NPoM structures are further covered with a thin film of poly(methyl methacrylate) (PMMA).^42^ This coating with a high refractive index polymer (*n* = 1.49) enhances the coupling of light into the nanocavity and tunes the plasmonic resonance closer to the wavelength of anti-Stokes emission associated with the 1080 cm^–1^ and 1586 cm^–1^ vibrational modes (∼720 and 700 nm, respectively),^42^ allowing us to probe the anti-Stokes signal more reliably.

## Optomechanical Simulations

For the optomechanical modeling, we consider a Au nanosphere of 90 nm diameter truncated at the bottom by a circular flat facet of radius 16 nm and separated from the Au substrate by a 1.3 nm gap with dielectric constant ε_g_ = 2.1, as in previous works.^21^ The additional PMMA layer introduced in the simulations is 100 nm thick (see Supporting Information Section S5 for further details of the geometry). The simulated scattering cross section for this nanostructure (Figure 1d, solid black) shows radiative plasmonic modes at 660 nm and 890 nm. The resonance at 660 nm fits the experimentally observed dark-field spectrum (dashed black line) well, while the resonance at 890 nm may be related to an experimental peak outside the spectral range. This description of the plasmonic cavity sets up a robust basis to describe the Stokes emission. The radiative plasmonic modes are also seen in the simulated near-field enhancement |*E*_*z*_|/|*E*_0_| (Figure 1d, red), with *E*_0_ the amplitude of the incident electric field and *E*_*z*_ the amplitude of the *z*-component of the total electric field at 6 nm (radial distance) from the center of the cavity (see Supporting Information Section S5). A patch of molecules arranged in concentric rings (see Figure 5c and Supporting Information Section S5) is placed centered in this position in optomechanical simulations, since higher field enhancement is observed there compared to the center of the facet due to the symmetry of the excited modes and to illumination conditions (see Supporting Information Figure S8 for field profiles at resonance frequencies). We use a maximum of 217 molecules in the simulation, corresponding to intermolecular distances *d* ≈ 0.6 nm. The plasmonic modes and the plasmon-mediated intermolecular interactions are included in the optomechanical model via the near-field enhancement and the scattering dyadic Green’s function , which are obtained numerically using the finite element method solver COMSOL Multiphysics.^43^ In contrast to the field enhancement, the self-interaction Green’s function  of the NPoM (Figure 1e) carries information also of the nonradiative plasmonic modes. We observe that the self-interaction Green’s function is dominated by a strong pseudomode at λ_PPM_ ≈ 520 nm as previously reported,^21^ showing that the PMMA coating does not alter significantly the nonradiative modes of the plasmonic cavity.

We employ the general molecular optomechanics model introduced in ref (21) to describe the vibrational dynamics under continuous-wave (CW) illumination, where the collective effects of many molecules are taken into account, as well as the full plasmonic response via the Green’s function  of the NPoM. This model does not account for the influence of intramolecular vibrational redistribution,^15,35,44,45^ which we expect to affect more importantly the low-wavenumber vibrational modes. In Section S10 of the Supporting Information, we show that this CW model can be used to describe the experiments in this work, which are conducted with pulsed illumination, after an appropriate scaling of the vibrational decay rate. We consider the case of identical molecules, i.e. with the same vibrational frequency ω_ν_ and decay rate γ_ν_. The dynamics of the population  of the molecule ν and of the correlation  between the molecules ν and ν^′^ is given by
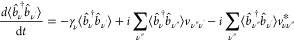
1where  accounts for the coupling between the molecules ν and ν′ due to the optomechanical interaction. Here,
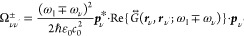
2is responsible for the optomechanical spring shifts induced in the vibrational frequencies by the plasmonic response of the nanocavity acting as a reservoir,^18,21^ while
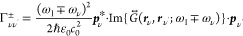
3introduces the modification of the vibrational decay rates. We have introduced in eqs 2 and 3 the molecular position ***r***_ν_ and the induced Raman dipole ***p***_ν_ (given by the near-field enhancement and the Raman tensor^18^), as well as the speed of light in vacuum *c*_0_, the vacuum permittivity ε_0_, the Planck constant ℏ and the laser frequency ω_l_. Γ_νν_^–^ and Γ_νν_^+^ represent the modification of the vibrational damping and pumping rates, respectively, of single molecule vibration ν due to the plasmonic response of the nanocavity.^19,46^ Thus, the resulting decay rate of the vibrational mode of each molecule is , with Γ_νν_^opt^ = Γ_νν_^–^ – Γ_νν_^+^ the optomechanical damping rate. As an example, we plot in Figure 1f the rates Γ_νν_^–^ and Γ_νν_^+^ for the vibrational mode ω_ν_ = 1586 cm^–1^ of a molecule situated at the center of the patch, which show two peaks at the wavelengths of the radiative plasmonic modes. Γ_νν_^opt^ is positive over almost all of the spectral range shown in Figure 1f (indicating Γ_νν_^–^ > Γ_νν_^+^) and has a maximum at 650 nm. This behavior of Γ_νν_^opt^ contrasts with the results of a simpler model that considers only one vibrating molecule and a single Lorentzian plasmonic resonance,^46^ where a laser that is blue-detuned with respect to the plasmonic resonance always results in a decrease of the effective vibrational decay rate (Γ_νν_^opt^ < 0), whereas a red-detuned laser leads to optomechanical damping (Γ_νν_^opt^ > 0). Therefore, in this NPoM system for the molecular vibration at 1586 cm^–1^, the system is effectively mainly in the red-detuned optomechanical regime preventing the observation of certain optomechanical effects such as parametric instability.

Using eq 1, the steady-state of the vibrational modes and hence the Stokes (S) and anti-Stokes (aS) signal can be obtained. The calculation of the Stokes and anti-Stokes differential scattered power is based on the formalism of collective vibrational modes introduced in ref (21), which yields
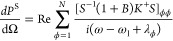
4
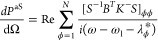
5where the summation is over the *N* collective modes with index ϕ. *B* represents the matrix of the steady-state vibrational populations and correlations, with elements  (where subscript ss indicates the steady-state) and *K*^±^ are the Stokes and anti-Stokes propagator matrices, with elements
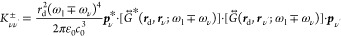
6where ***r***_d_ is the position of the detector. Additionally, the matrix *S* in eqs 4 and 5 corresponds to the eigenvectors of the matrix  so that *S*^–1^MS = diag{λ_1_,λ_2_,···,λ_*N*_} correspond to the eigenfrequencies. The contribution of each molecule *v* to the collective mode ϕ is given by *S*_ϕν_. Equations 4 and 5 decompose the Raman signal into the contribution of each collective molecular vibrational mode and thus enable one to identify the different bright and dark vibrational collective modes, as well as their line widths and spring shifts (see Supporting Information Section S9).

## Power-dependent SERS Experiments

To investigate the molecular optomechanics response of the plasmonic-molecular system experimentally, we perform laser-power dependent SERS spectroscopy. Using picosecond laser pulses, we are able to excite the structures with high peak powers while average powers stay below the threshold for damage to the molecules and nanostructures. This allows probing of the nonlinear optomechanical regime usually not accessible with continuous wave (CW) illumination. For these experiments, we use 785 nm laser pulses with 0.5 ps duration at 80 MHz repetition rate. At 2 nm bandwidth, such pulses give sufficient spectral resolution to measure individual Raman lines while still achieving high peak powers. Average laser powers  are varied from 500 nW to 20 μW with integration times scaling inversely with power (200 s integration at 1 μW) to achieve comparable signal-to-noise at low powers and limit damage at high powers. Using notch filters, both Stokes and anti-Stokes scattered light from the nanocavity can be recorded with a spectrometer. Experiments are carried out on hundreds of NPoMs using automated particle tracking algorithms to locate and center each nanostructure in the laser focus. To compare the results of the experiments to optomechanical simulations, average laser powers are converted to peak laser intensities. Here, an average power  of 1 μW corresponds to a peak intensity *I*_l_ of 8.9 × 10^4^ μW μm^–2^ in the laser focus (see Methods).

**Figure 2:**
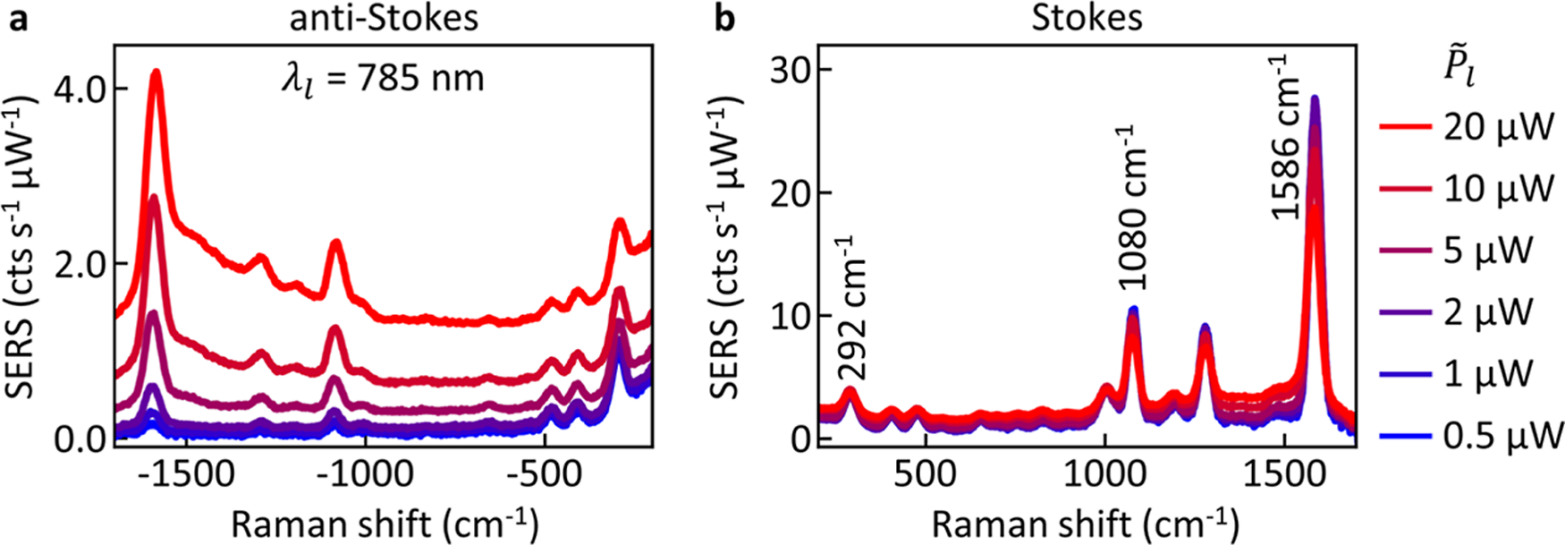
Power-dependent experimental pulsed SERS spectra. (a) Power-normalized anti-Stokes and (b) Stokes SERS spectra of BPT in NPoM nanocavities, excited with a pulsed laser (785 nm, 80 MHz, 0.5 ps) of average power  ranging from 0.5 to 20 μW (colors). Spectra are sorted by coupling-corrected laser power, averaged over 70 NPoMs, and normalized by integration time and excitation power. Vibrational modes investigated in Figure 3 are labeled in (b).

We obtain power-dependent SERS spectra from averaging over 70 individual nanostructures and normalize by integration time and average laser power (Figure 2). In this normalization, constant SERS spectra indicate a linear scaling proportional to the laser power, as seen for the Stokes spectra. The anti-Stokes spectra instead exhibit a strongly superlinear scaling, with both normalized SERS peaks and background increasing with laser power (Figure 2a). The superlinear increase of anti-Stokes SERS lines (with higher-energy vibrations showing a stronger effect than lower-energy vibrations) indicates a rise in the vibrational population and will be analyzed in more detail below. On the other hand, the increased SERS background is attributed to electronic excitations. Such broadband signals from electronic Raman scattering (ERS) are studied in more detail in Supporting Information Section S1. While the background at low wavenumbers indicates the temperature of a thermalized population of electrons,^47,48^ nonthermalized (“hot”) electrons lead to an almost constant background signal stretching to high wavenumbers.^49^ With higher laser power, a larger population of hot electrons is excited and the temperature of thermalized electrons increases.

In contrast to the anti-Stokes signal, Stokes spectra scale linearly with laser power for most of the powers investigated here (Figure 2b). Only at the highest laser powers does the area of SERS lines reduce, while the background which is red-detuned from the SERS lines increases. This effect was previously attributed to the optomechanical spring shift redistributing the vibrational energy, red-shifting and broadening the vibrations. This shift leads to weakening of the main SERS peak and a nonlinear increase of the background red-shifted to the vibrational mode.^21^ Here, we also observe the occurrence of a shoulder in anti-Stokes spectra to the lower energy side of the 1586 cm^–1^ mode increasingly visible at higher laser powers (Figure 2a). This observation provides further evidence that the optical spring effect in this molecular optomechanical system can lead to vibrational energy shifts >100 cm^–1^, and is consistent with previous experiments.^21^ In the following, we focus on the analysis of the vibrational pumping.

From individual SERS spectra of each NPoM, SERS intensities of multiple vibrational modes are extracted by fitting a Gaussian peak after background subtraction. Comparing the results of different NPoMs is however difficult since the light is coupled from the far field to the nanocavity with varying efficiency due to variations in nanoparticle size and contact facet shape.^50,51^ We account for these variations through renormalizing the laser intensities *I*_l_ with the coupling efficiency (which includes in- and out-coupling of light from the far-field to the near-field) of each NPoM (indexed by *i*) by calculating the coupled laser intensities *I*_*i*_^c^ = η_*i*_*I*_l_. Each coupling efficiency η_*i*_ can be estimated using the SERS signal *S*_*i*_ at the lowest laser intensity using η_*i*_ = *S*_*i*_/mean(*S*_*i*_) assuming linear scaling of the signal with power (*S*_*i*_ ∝ *I*_l_). When quadratic scaling of the signal with laser power is expected (*S*_*i*_ ∝ *I*_l_^2^), the coupling efficiency is given by . Since each vibrational line leads to scattering at a different wavelength and field enhancement depending on the NPoM plasmonic resonances, the coupling efficiency is calculated separately for each vibrational mode and for Stokes and anti-Stokes signals.

**Figure 3:**
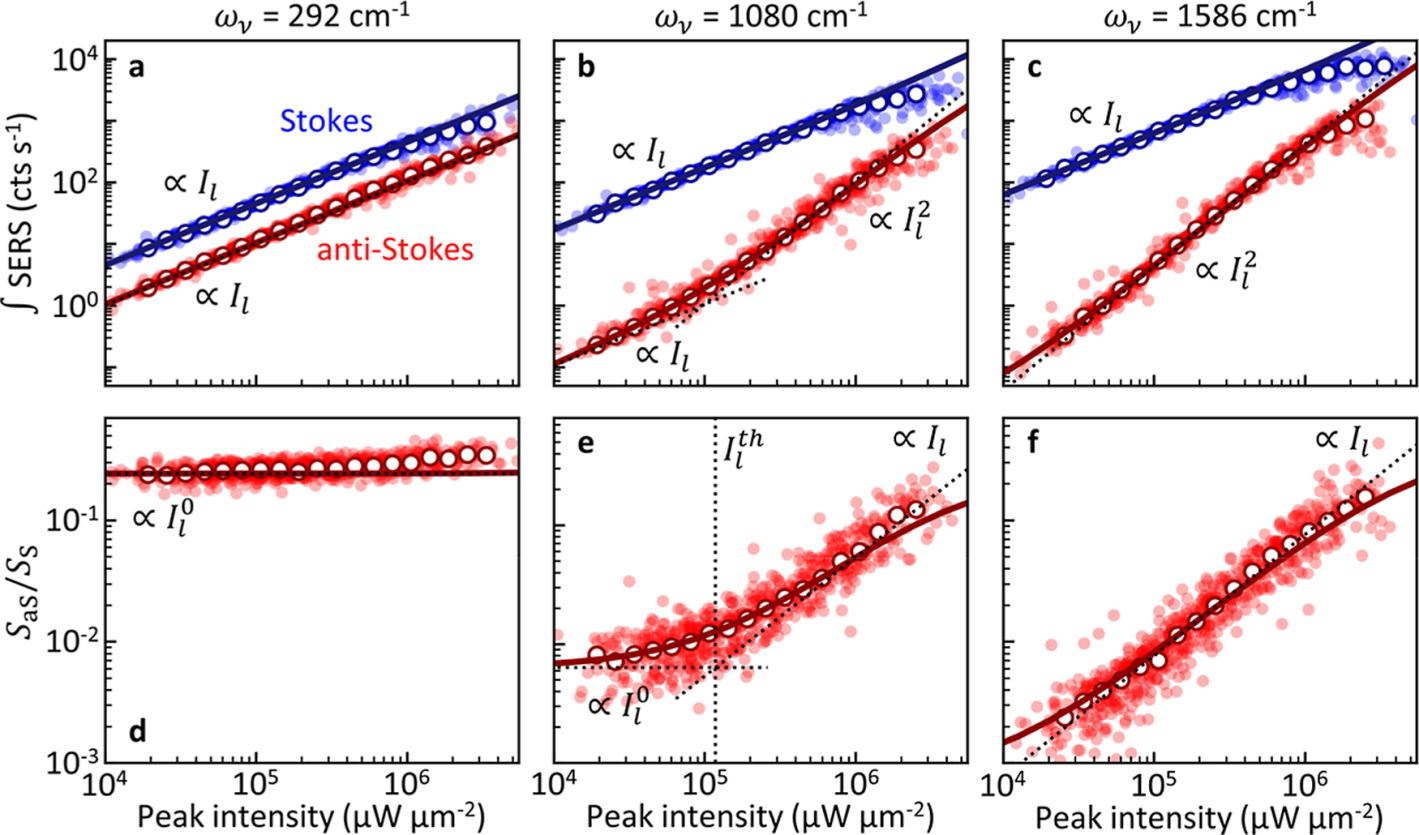
SERS signal vs laser intensity. (a–c) Extracted Stokes (blue) and anti-Stokes (red) signal vs coupling-corrected peak intensity of the pulsed excitation laser (785 nm, 80 MHz, 0.5 ps) for (a) 292 cm^–1^, (b) 1080 cm^–1^ and (c) 1586 cm^–1^ vibrational modes. Linear dependence of anti-Stokes signal on laser intensity in the thermal regime changes to quadratic dependence in the vibrational pumping regime. (d–f) Anti-Stokes to Stokes signal ratio for the (d) 292 cm^–1^, (e) 1080 cm^–1^ and (f) 1586 cm^–1^ vibrational modes. Filled points are experimental data from 70 individual NPoMs, open circles are averages of all particles. Solid lines are simulations with the optomechanical model for a patch of 217 molecules and CW illumination at 785 nm wavelength, dotted lines show the indicated scaling law. The simulated signal in (a–c) is obtained in arbitrary units and scaled by a constant factor for better comparison. The simulated anti-Stokes to Stokes ratios in (d–f) are scaled by factors 0.8, 1.2 and 0.9, respectively. Additionally, the intensity used in the simulations in (b,e) is scaled by a factor 0.2 to match with the experimental threshold intensity (see main text).

To investigate the power-dependent behavior of modes with different vibrational energies, we focus on the most evident vibrations at ω_ν_ = 292 cm^–1^, 1080 cm^–1^ and 1586 cm^–1^ (Figure 3). While the lowest energy mode exhibits a high thermal population at room temperature for the range of *I*_l_ considered, the vibrationally pumped population dominates for the highest energy vibration. For the 292 cm^–1^ vibration, both Stokes and anti-Stokes signals scale linearly with laser intensity *I*_l_ (Figure 3a). This scaling indicates that the population remains approximately constant, and thus that the sample is not significantly heated (Supporting Information Section S2). The 1586 cm^–1^ vibration on the other hand shows quadratic scaling of the anti-Stokes signal with *I*_l_ while the Stokes signal is proportional to *I*_l_ (Figure 3c). As laser heating has been excluded, this scaling is clear evidence for vibrational pumping of the phonon population by the surface-enhanced Stokes scattering rate (see also Supporting Information Section S2). We note that at high laser intensities the experimentally observed signal saturates which has previously been attributed to the optomechanical spring effect red-shifting and broadening the vibrational line.^21^ Interestingly, the transition from the thermally dominated to the vibrational pumping regime can be observed for the 1080 cm^–1^ vibration (Figure 3b). This is evidenced by a transition from linear to quadratic scaling of the anti-Stokes signal (dotted lines), with the latter characteristic of the vibrational pumping regime. Here, the transition occurs at intensity *I*_l_^th^ ≈ 10^5^ μW μm^–2^. Below, we will investigate the influence of collective vibrations on vibrational pumping by analyzing changes with intermolecular distance in the threshold intensity where this transition occurs. For the other two vibrational modes investigated, the intensity of this transition is either above (292 cm^–1^) or below (1586 cm^–1^) the accessible laser intensity range in this experiment.

The optomechanical model predicts the experimental scaling of the SERS signal with laser intensity well (solid lines in Figure 3a–c, obtained for 217 molecules and illumination wavelength 785 nm). The SERS signal from optomechanical simulations was scaled with a free fitting parameter to match the experimental units and account for collection efficiencies of the setup. Additionally, to reproduce the intensity threshold for vibrational pumping of the 1080 cm^–1^ mode, laser intensities from the simulation needed to be scaled by a factor 0.2. This implies that in the simulation, vibrational pumping is underestimated and laser intensities 5-fold lower than predicted are required in the experiment to reach the vibrational pumping regime. The main contributions to this scaling factor are likely that the simulation considers a smaller number of molecules than present in the experiment (among other differences between the exact experimental and theoretical configuration), as well as the influence of nonlocal effects and the potential underestimations of the coupling efficiency, the Raman tensor of this mode, and the optomechanical coupling between molecules. Taking into account the difficulty of exact quanti-tative agreement with SERS measurements, we consider this relatively small discrepancy as very satisfactory. Further, we discuss below how the agreement between theory and experiment becomes much worse if the collective interactions are ignored, firmly supporting the key role of collective vibrational modes in the emission process. For the 292 cm^–1^ and 1586 cm^–1^ vibrations, no scaling of the powers is used as no threshold is observed that can be determined for calibration.

In Raman scattering experiments, the anti-Stokes/Stokes signal ratio (*S*_aS_/*S*_S_) is commonly used as a measure for the vibrational population or local temperature.^52,53^ In Figure 3d–f, we show *S*_aS_/*S*_S_ for the three vibrational modes. Again, the regime with thermally dominated population (constant *S*_aS_/*S*_S_) can be clearly distinguished from the vibrational pumping regime (*S*_aS_/*S*_S_ ∝ *I*_l_). Remarkably, the optomechanical model reproduces the experimentally measured ratio for 292 cm^–1^, 1080 cm^–1^ and 1586 cm^–1^ well with small correction factors scaling the simulated intensity ratios by 0.8, 1.2 and 0.9, respectively. Again, the intensities of simulations for the 1080 cm^–1^ mode were scaled by 0.2 to match the threshold intensity. This shows that our simulations including many molecules capture the ratio of field enhancements at the Stokes and anti-Stokes wavelengths well for the NPoM structures. At high laser intensities, the optomechanical simulation predicts deviations from linear scaling which are difficult to verify in this experiment as molecular damage occurs at these intensities (likely linked to strong vibrational pumping). In brief, the generally good agreement between the theoretical and experimental Raman signal and *S*_aS_/*S*_S_ ratio indicates that the laser induces vibrational pumping in high-wavenumber vibrational modes, and that the latter is enhanced by the collective interactions. We remark that it is also conceivable that other effects, such as hot electrons, internal vibrational redistribution, or fluorescence pumping,^15^ may give additional contributions to the increase of the vibrational population.

Assuming a single collective vibrational mode coupled to a single cavity mode (the limitations of these approximations are discussed below), the collective vibrational population can be estimated from the *S*_aS_/*S*_S_ ratio with the classical model of Raman scattering.^8^ In Supporting Information Section S2, we calculate this collective vibrational population and develop a simple analytical model for vibrational pumping. By comparing this model to a model of laser-induced heating, we conclude that the 1080 cm^–1^ and 1586 cm^–1^ modes are dominantly excited by vibrational pumping as expected, while the lower-energy vibrations are likely populated by local heating of the sample by the laser or other mechanisms (see, for instance, the small increase of *S*_aS_/*S*_S_ at high laser intensities in Figure 3d, which contrasts with the constant value obtained with the vibrational pumping simulations).

Moreover, we demonstrate that the use of pulsed lasers is necessary to investigate vibrational pumping in our experimental system by carrying out power-dependent SERS measurements with a continuous-wave (CW) laser (see Supporting Information Section S3). Here, linear scaling for both Stokes and anti-Stokes signal is observed for all vibrational modes, and hence vibrational populations are not excited above their thermal population. This result is expected since intensities achieved with the CW laser (higher CW intensities irreversibly damage samples) are 3 orders of magnitude below the threshold intensities *I*_l_^th^ found with pulsed experiments.

## Collective Molecular Vibrational Coupling

**Figure 4:**
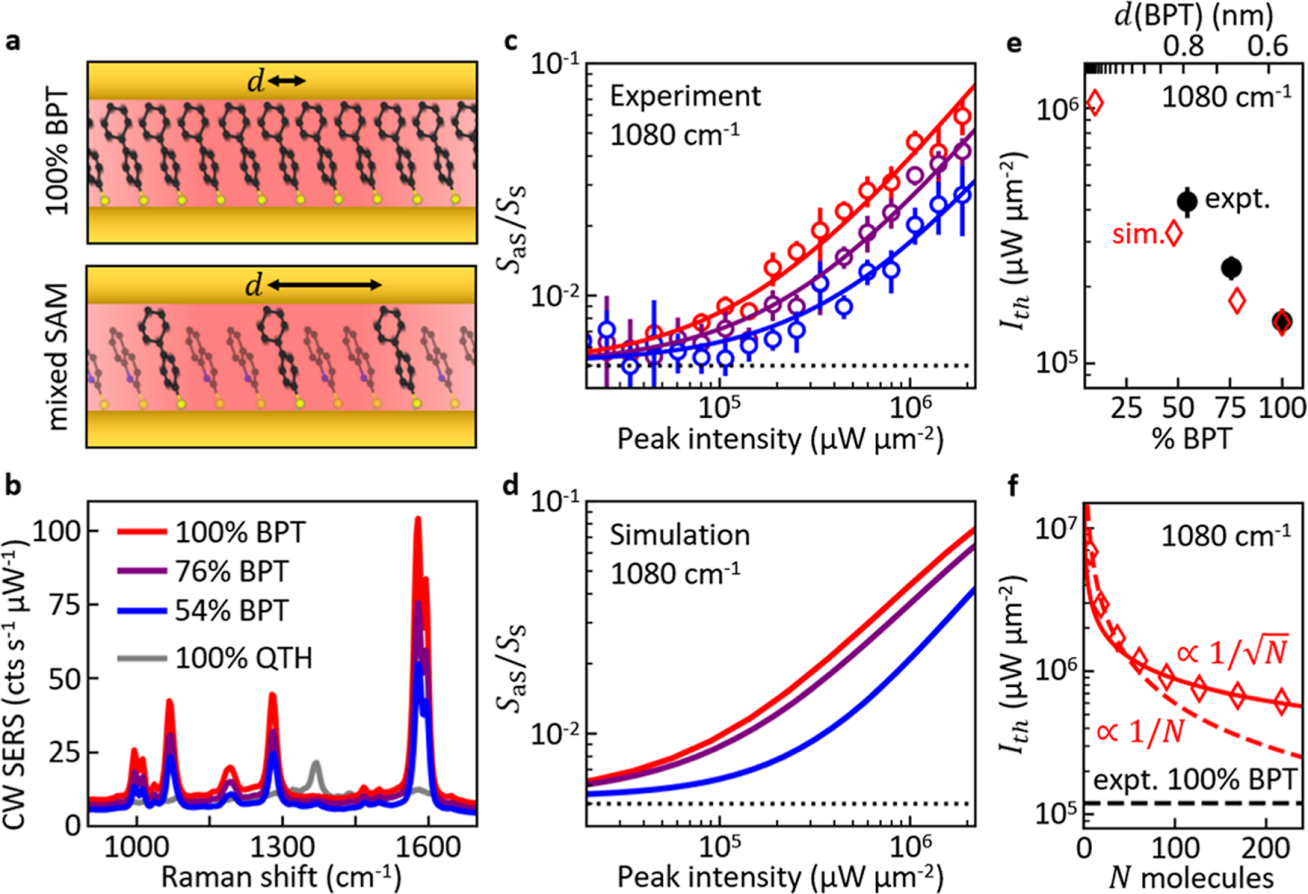
Collective vibrational pumping. (a) Mixed monolayers of BPT and QTH allow experimental changes of effective BPT intermolecular spacing *d* to tune collective coupling. (b) Average CW Raman spectra from ∼100 NPoMs for SAMs prepared with different ratios of BPT to QTH. BPT coverage in SAMs is estimated from average BPT SERS signal from the 1080 cm^–1^ vibrational mode. (c) Experimentally measured anti-Stokes to Stokes signal ratio with laser intensity (pulsed illumination, wavelength 785 nm, 80 MHz, 0.5 ps), for different molecular dilutions, of BPT of the 1080 cm^–1^ vibrational mode (dotted line indicates ratio in the thermal regime). Open circles are experimental averaged data from many NPoMs, solid lines are numerical fits. Higher concentration of BPT increases vibrational pumping rates, indicating stronger collective effects. Circles indicate averages of many NPoMs with error bars showing standard errors. See (b) for labels. (d) Simulated anti-Stokes to Stokes signal ratio under 785 nm wavelength CW illumination for intermolecular distance *d* ≈ 0.6 nm (red), 0.68 nm (purple), 0.85 nm (blue), corresponding to *N* = 217, 169, 113 molecules (chosen to match closely the experimental effective distance). The intensity is scaled by a factor 0.2 to match with the experimental threshold intensity. (e) Intensity threshold *I*_th_ for vibrational pumping of the 1080 cm^–1^ mode for different BPT dilutions from experiment (black dots) and optomechanical simulation (red diamonds, scaled to match experiment at 100% BPT). The results are plotted as a function of the estimated experimental fraction of BPT in the SAM, and of the average intermolecular distance in the calculations. (f) Intensity threshold for vibrational pumping (red diamonds, not scaled) vs number of molecules in the gap in the simulation, for *d* ≈ 0.6 nm (100% BPT). The experimentally observed threshold for a 100% BPT sample is indicated (dashed black line). Fits for scaling of *I*_th_ with *N* are indicated for  (solid red line) and ∝1/*N* (dashed red line).

We further investigate how collective vibrations influence vibrational pumping in the NPoM system. Previous experiments have established that it is possible to increase the spacing between molecules in the SAM by mixing them with another species of molecule with a distinctly different vibrational spectrum (Figure 4a).^20,54^ With this approach, it was demonstrated that IR-active vibrations form a collective state by IR transition dipole–dipole coupling which leads to a frequency shift of the vibration in the Raman and IR spectra.^20,54,55^ Here instead, we study the optomechanical coupling of induced Raman dipoles through the plasmonic response of the nanocavity. In comparison to the vibrational IR transition dipoles oscillating at mid-IR frequencies ω_ν_, the induced Raman dipoles oscillate at visible/near-IR frequencies ω_*S*_ and ω_aS_.

To tune the effective spacing of BPT molecules in the SAM, mixed molecular layers are prepared by adding 2-quinolinethiol (QTH) with different concentrations (Figure 4a). QTH was chosen since its SERS spectrum (gray in Figure 4b) does not overlap with the vibrational modes under investigation in BPT (red in Figure 4b), in particular the 1080 cm^–1^ mode. Additionally, the aromatic structure of QTH ensures good mixing with BPT in the SAM without the formation of domains, see below. Solutions with molar mixing fractions 100:0, 50:50, 10:90 and 0:100 of BPT:QTH are prepared and used for NPoM sample fabrication. We note that Au mirror substrates are here coated with a monolayer of Pd atoms before SAM formation to suppress the occurrence of picocavities which cause strong intensity fluctuations of the SERS signal.^58^ To characterize the actual molecular fractions of the mixed SAMs, we record continuous-wave SERS spectra of >100 NPoMs for each sample. The average spectra of all particles are shown in Figure 4b. From the intensity of BPT 1080 cm^–1^ SERS, we determine the BPT fraction of the SAMs to be 76% and 54% ± 4% for the 50:50 and 10:90 mixing in solution, respectively (see Supporting Information Figure S6c). This shows that BPT has a higher binding affinity to the substrate than QTH and higher dilutions would be needed to form a sparser layer of BPT. Further, we confirm homogeneous mixing of the two molecules in the SAM by measuring the ratio of the QTH 1320 cm^–1^ to BPT 1080 cm^–1^ peaks (see Supporting Information Figure S6a,b). While the QTH signal is much weaker than BPT, the histogram of all NPoMs is narrow and shows clear differences in the ratios for different mixing fractions during sample preparation. This confirms that both molecules mix well and do not form domains on the length scale ∼10 nm probed by NPoM cavities (i.e., the molecular environment under each NPoM is similar). Hence, we conclude that BPT and QTH form homogeneous mixed SAMs and this method is suitable to investigate collective effects in molecular optomechanics.

To investigate how vibrational pumping is changed by the increased intermolecular separation, power-dependent SERS measurements under pulsed illumination are again carried out as described above. The intensity-dependent *S*_aS_/*S*_S_ ratio for the 1080 cm^–1^ mode is extracted for ∼100 individual NPoMs on each sample, corrected for coupling efficiency, and averaged. The threshold for vibrational pumping is pushed to higher laser intensity when BPT is diluted and hence the intermolecular spacing increases (Figure 4c). The same effect is observed in optomechanical simulations when molecules are spaced out further (keeping the area of the molecular patch constant and thus reducing the number of BPT molecules with increasing distance, analogously to experiments with higher dilution, see Supporting Information Section S8 for further information) (Figure 4d). A patch of *N* = 217 molecules is considered for the shortest intermolecular distance, *d* ≈ 0.6 nm, corresponding to no dilution. To quantify the threshold intensity for vibrational pumping *I*_th_, the *S*_aS_/*S*_*S*_ ratio from both experiment and simulation is fit with a constant and a linear term, and *I*_th_ is determined from the intersection of both terms (this is analogous to the analytical model for vibrational pumping developed in Supporting Information Section S2). The values obtained are plotted in Figure 4e. A 3-fold increase of the intensity required for vibrational pumping is observed in the experiments at the highest dilution compared to the pure BPT SAM, corresponding to a 3-fold reduction of vibrational pumping rates due to the reduced intermolecular coupling. The optomechanical model shows a similar trend although it does not reproduce the magnitude of the effect fully. This discrepancy may be due to mis-calibrations in measuring the actual concentration of BPT in the monolayers, or possible additional disorder of the molecules in the mixed SAM. Nevertheless, the theoretical modification of the vibrational pumping rate with intermolecular distance is in general agreement with the experiments, providing clear experimental evidence for the importance of collective vibrations in SERS from NPoM nanocavities.

Additionally, we further investigate the collective response in our simulation. By varying the size of the patch within the cavity from *N* = 1 to 217 molecules with *d* ≈ 0.6 nm, we track the influence of collective coupling on the vibrational pumping threshold of the 1080 cm^–1^ mode. Increasing the number of molecules *N* and thereby enhancing the collective optomechanical coupling, the vibrational pumping rate is enhanced and the threshold intensity hence decreases  for large *N* (see Figure 4f, note that the simulated thresholds in this figure are not scaled to match the experiment). If the  scaling is robust, approximately *N* ≈ 5000 molecules would be needed in the simulation to match the threshold observed in experiments (which is larger than the maximum number of molecules that can fit underneath an NPoM facet, ≈2100). This result should be taken with care due to extrapolation of the scaling by almost one order of magnitude, but it emphasizes the general agreement between experiment and theory.

The scaling factor used to coalign experiment and simulations could therefore mainly be the consequence of our model not utilizing the required number of molecules (due to prohibitive computational requirements). As mentioned above, other potential sources of error include the calculation of Raman tensors from DFT (see Supporting Information Section S7) and the coupling efficiency of the plasmonic nanostructure. The strong dependence of the threshold intensity on the number of molecules in the simulation however emphasizes the importance of including collective vibrational coupling in the optomechanical modeling. Simulating a single molecule, we obtain a threshold intensity ≈ 4 × 10^7^ μW μm^–2^, two orders of magnitude larger than in the experiment. A similar value is obtained ignoring collective effects and treating the molecules as independent.

## Collective Coupling and Vibrational Populations

As mentioned above, vibrational populations and effective temperatures are often extracted in experiments from the anti-Stokes to Stokes ratio *S*_aS_/*S*_S_. However, the usual procedure does not consider collective effects. Within the optomechanical framework, the *S*_aS_/*S*_S_ ratio from a single molecule or from an arbitrary number of independent molecules (that couple identically with the plasmonic mode) is given as^19,46^
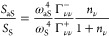
7where *n*_ν_ is the vibrational population of each molecule ν (identical for all molecules). Γ_νν_^–^ and Γ_νν_^+^ account for the modification of the driving of Stokes and anti-Stokes processes, respectively, induced by the plasmonic response of the nanocavity (see eq 3). Solving for the vibrational population in eq 7 yields
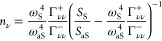
8The parameters Γ_νν_^–^ and Γ_νν_^+^ cannot be easily obtained from experiments. Neglecting the Γ_νν_^+^/Γ_νν_^–^ factor inside the parentheses in eq 8, it is possible to define an “effective” population as
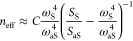
9where we have replaced the factor Γ_νν_^+^/Γ_νν_^–^ by a proportionality constant *C* outside the parentheses to emphasize that, in experiments, this factor can be treated as a correction obtained by matching the experimental and thermal population at weak enough laser intensity. Ignoring this constant *C* can lead to erroneous estimations of the populations.^32,33,56,57^

The expression of the anti-Stokes to Stokes ratio however becomes more intricate when collective effects are considered. From the integral of eqs 4 and 5, we obtain
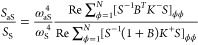
10which shows that the population of all collective modes ϕ, , contributes to the ratio. Thus, in general not only the vibrational population of each molecule  needs to be taken into account, but also the correlations  between the molecules (i.e., the molecular interactions). Therefore, the physical meaning of *n*_eff_ extracted from eq 9 is in principle ill-defined when collective effects are present. We investigate next to what extent this effective population is connected to the simulated population of different collective modes.

**Figure 5:**
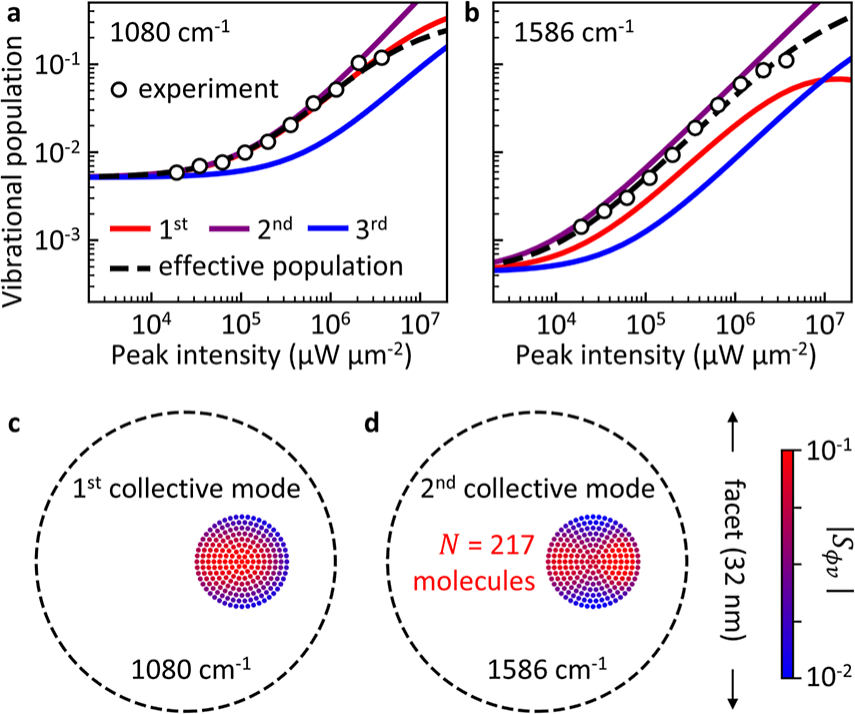
Collective molecular vibrational modes. (a,b) Simulated vibrational populations of the first ϕ = 1 (red), second ϕ = 2 (purple) and third ϕ = 3 (blue) collective mode as a function of the excitation laser intensity for the (a) 1080 cm^–1^ and (b) 1586 cm^–1^ vibrational modes. The black dashed line indicates the simulated effective vibrational population calculated from *S*_aS_/*S*_S_ according to eq 9, while black circles indicate the effective population measured in the experiment (see Figure 3). The experimental population in (a) is scaled to match thermal population at room temperature for low laser intensities, while in (b) it is scaled to match the simulated effective population. Additionally, the intensity used in the simulations in (a) is scaled by a factor 0.2 to match with the experimental threshold intensity (see main text). (c,d) Simulated spatial distribution of the collective vibrational modes (c) ϕ = 1 and (d) ϕ = 2 for the molecular patch consisting of 217 molecules and shifted 6 nm from the center of the gap. The color of each molecule *v* indicates its contribution |*S*_ϕν_| to the collective mode ϕ at *I*_l_ = 1.2 × 10^7^ μW μm^–2^ and laser wavelength 785 nm, for the (c) 1080 cm^–1^ and (d) 1586 cm^–1^ vibrational modes of the individual molecules.

We consider a patch of *N* = 217 molecules in the NPoM cavity at CW laser wavelength 785 nm, considering the same modification of the vibrational decay rate (see Supporting Information Section S10). Figure 5a,b show the dependence on the laser intensity of the populations *n*_ϕ_ of the collective modes ϕ = 1 (red), ϕ = 2 (purple) and ϕ = 3 (blue) and of the effective population *n*_eff_ (black) for the (c) 1080 cm^–1^ and (d) 1586 cm^–1^ vibrational modes. *n*_eff_ is obtained applying eq 9 to the simulated *S*_aS_/*S*_S_. In the case of the 1080 cm^–1^ vibrational mode, the contributions of the collective mode ϕ = 1 dominate both the numerator (anti-Stokes signal) and denominator (Stokes signal) in eq 10 (despite the larger population of the collective mode ϕ = 2). Consequently, the effective population in Figure 5a agrees well with the population of this collective mode ϕ = 1. To characterize the spatial distribution of this collective mode which dominates the Raman signal, we plot in Figure 5c the contributions |*S*_ϕν_| to the ϕ = 1 mode from each molecule ν at *I*_l_ = 1.2 × 10^7^ μW μm^–2^. We find that this mode exhibits radial symmetry with slight distortions due to the complex plasmonic modal structure and the inhomogeneous spatial distribution of the electric field in the gap (see Supporting Information Section S6).

For the 1586 cm^–1^ vibrational mode, the contributions of the collective modes ϕ = 1 and ϕ = 2 to the numerator and denominator in eq 10 are comparable. In this case, the effective population falls between the populations of the ϕ = 1 and ϕ = 2 collective modes (Figure 5b). The spatial distribution of the ϕ = 1 collective mode is again similar to that shown in Figure 5c, while the spatial distribution of the collective mode ϕ = 2 shows two lobes on opposite sides (Figure 5d). A more complete characterization of the collective modes, including line widths and optomechanical spring shifts, is provided in Supporting Information Section S9.

Comparing the simulated effective population with the population extracted from the experimentally measured *S*_aS_/*S*_S_ we find good agreement between our model and the experiment (Figure 5a,b). To account for the factor *C* in eq 9, the experimental populations were scaled with factors 1.6 and 1.8 for the 1080 and 1586 cm^–1^ modes, respectively. As previously discussed, the laser intensity in simulations of the 1080 cm^–1^ mode is again scaled by 0.2 to match the observed threshold intensity for vibrational pumping.

More generally, the analysis in Figure 5 highlights that the effective vibrational population typically measured in SERS through the *S*_aS_/*S*_S_ ratio may not represent the populations of the collective vibrational modes well. While the measured effective population follows the ϕ = 1 collective mode well for the 1080 cm^–1^ vibration, it does not represent exactly any of the collective modes for the 1586 cm^–1^ vibration. Other configurations may lead to even larger disagreement. Thus, we conclude that although the effective population can contain useful information about the population of the collective modes, the results need to be interpreted carefully in the context of optomechanical simulations.

## Conclusion

In this paper, we have investigated the laser power dependence of Stokes and anti-Stokes scattering of molecules in NPoM cavities. Analyzing data from several hundreds of individual nanostructures, we have shown that superlinear scaling of the anti-Stokes signal indicates vibrational pumping by the pulsed laser illumination under ambient conditions. Moreover, by tuning the spacing between BPT molecules using mixed SAMs, we have presented experimental evidence that intermolecular vibrational coupling significantly affects the magnitude of optomechanical effects. To simulate these experimental results, we have developed a model based on molecular optomechanics accounting for the full multimode plasmonic response in a continuum-field model. Additionally, to fully capture the magnitude of vibrational pumping it is essential to model the collective vibrational response by calculating the full response, here for >200 coupled vibrational dipoles in the cavity. Our experiments and theoretical calculations are generally in very satisfactory agreement, offering further support to the identification of quadratic scaling of the anti-Stokes signal with vibrational pumping, and to the optomechanical origin of this vibrational pumping. Both experiments and simulations hence indicate that the optomechanical interaction leads to the emergence of collective vibrational modes in highly ordered molecular layers in plasmonic nanocavities. Additionally, the occurrence of collective molecular vibrations implies synchronized oscillation of many molecules in this tightly confined system. This coherence should be taken into consideration when interpreting time-resolved vibrational spectroscopy experiments where molecular vibrations are excited with coherent Raman scattering.^58^ Understanding and manipulating the molecular optomechanics of the coupled molecule-plasmon system could be essential to develop applications in plasmon-driven chemistry, phonon lasing through optomechanical instabilities and beyond.

## Methods

### Sample Preparation

Template-stripped Au samples were prepared by evaporating a 100 nm thick Au film on a Si waver. The Au film was stripped off the Si waver with glass substrates (UQG Optics, UK) using UV glue (Norland 81 from Thorlabs), resulting in a flat Au surface. Monolayers of biphenyl-4-thiol (BPT, Sigma-Aldrich) were prepared by placing the freshly stripped Au substrates into a 1 mM solution of BPT in 200-proof anhydrous ethanol overnight. The samples were washed with ethanol and blow-dried with nitrogen. To fabricate NPoM cavities, 40 μL of 80 nm Au nanoparticles (BBI Solutions) mixed with 0.1 M NaNO_3_ (10:1) were drop casted on the BPT-coated Au substate, washed off with DI water after 10 s, and blow dried with nitrogen. Finally, the samples were spin-coated with a 100 nm thick film of PMMA (950 k molecular weight, 2 wt % in anisole) by subsequently using 500 rpm for 10 s and 2000 rpm for 45 s. The samples were cured on a hot plate at 50 °C for 1 min to remove residual anisole solvent.

Mixed SAMs were prepared with the same steps as above by mixing 1 mM solutions of BPT and 2-quinolinethiol (QTH, Sigma-Aldrich) in 200-proof anhydrous ethanol with molar mixing fractions of 50:50 and 10:90 BPT/QTH. In all experiments with mixed SAMs (also including the pure SAM reference samples) the template-stripped Au samples were coated with a monolayer of Pd atoms. The Pd layer is deposited on the Au substrate using electrochemical underpotential deposition from 0.1 M H_2_SO_4_ + 0.1 mM H_2_PdCl_4_ aqueous solution. The coverage of Pd on Au is tuned to a single, complete layer by controlling the deposition charge while holding the potential at the underpotential deposition peak.^59^ NPoMs were prepared on the mixed SAMs as described above.

### Pulsed SERS Spectroscopy

In a custom-built inverted darkfield microscope, NPoM nanocavities are located automatically by particle tracking algorithms and centered moving the sample stage. On each NPoM nanocavity, Raman and dark-field spectra are acquired in quick succession. Dark-field spectra are compared before and after laser illumination to ensure that no damage to the nanostructure was incurred. For pulsed Raman spectroscopy, laser pulses at 785 nm with 2 nm bandwidth and 0.5 ps duration are prepared by filtering the output of a Spectra-Physics Maitai pulsed laser with a tunable bandpass filter (80 MHz repetition rate). Laser pulses are focused on the sample by a 0.9 NA, 100× dark-field objective, which also collects the light scattered by the sample. The SERS signal is filtered by 785 nm notch filters and recorded with an Andor iDus DU416A camera mounted on an Andor Shamrock monochromator with a grating with 600 lines/mm.

For power-dependent SERS experiments, the average laser power is varied over 2 orders of magnitude with a variable neutral density filter. To achieve comparable signal-to-noise at all laser powers and avoid damage by laser pulses at high powers, integration times are scaled inversely with laser power to keep constant fluence. In general, the shortest possible exposure times are chosen to prevent damage. In each experiment, we record thousands of SERS spectra by examining hundreds of individual NPoMs for long periods of time.

### Analysis of SERS Spectra

SERS spectra are corrected for the spectral transmission efficiency of the optical setup and detection efficiency of the spectrometer camera with a broadband light source of known spectrum to allow measurement of the anti-Stokes to Stokes ratio. The SERS signals of individual Raman lines are extracted from SERS spectra by peak fitting. First, a high-order polynomial regression is performed to fit the spectral background without peaks. Then, the background-subtracted spectra are fit with multiple Gaussian peaks for each Raman line (line shapes are dominated here by the laser bandwidth and hence Gaussian). Finally, the integrated SERS signal of the Raman lines is obtained by calculating the area underneath each Gaussian fit. To compare the acquired signal intensities with theoretical calculations, the average laser power is converted to the peak intensity of the pulsed laser. For our laser pulses of 0.5 ps duration and 80 MHz repetition rate, an average power of 1 μW corresponds to a peak power of 2.5 × 10^4^ μW and a peak intensity of 8.9 × 10^4^ μW μm^–2^ for a laser focal spot of full width at half maximum ≈ 500 nm, which is close to the diffraction limit. The laser intensities are further corrected for each NPoM’s coupling efficiency as described in the main text.

## Data Availability

Data for all figures can be found at DOI 10.17863/CAM.116357.

## Supporting Information Available

Supplementary experimental data and analysis: electronic Raman scattering background; analytical models of vibrational pumping and laser heating; continuous-wave SERS; mixed SAM characterization. Supplementary theory and simulations: detailed description of simulations; characterization of the radiative plasmonic modes; DFT calculations of the Raman tensors; simulations with different molecular packing densities; contributions of the collective vibrational modes to the Raman signal; collective effects under pulsed illumination (PDF)
