# Supplementary material for: Optomechanical Pumping of Collective Molecular Vibrations in Plasmonic Nanocavities
Source: ACS Nano. 2025 Mar 14;19(11):10977–88. doi: 10.1021/acsnano.4c16535 (PMC11948455; doi:10.1021/acsnano.4c16535)
Supplement: Supplementary file 1 — nn4c16535_si_001.pdf [file nn4c16535_si_001.pdf]

Optomechanical Pumping of Collective Molecular Vibrations in Plasmonic Nanocavities

Supporting Information

Lukas A. Jakob[1†], Adrián Juan-Delgado[2,6†], Niclas S. Mueller[1,3 *], Shu Hu[1], Rakesh Arul[1], Roberto A. Boto[4], Ruben Esteban[2,4], Javier Aizpurua[4,5,6 *], Jeremy J. Baumberg[1 *]

[1] Nanophotonics Centre, Cavendish Laboratory, University of Cambridge, Cambridge CB3 0HE, UK.
[2] Centro de Física de Materiales (CFM-MPC), CSIC-UPV/EHU, Paseo Manuel de Lardizabal 5, 20018 Donostia-San Sebastián Gipuzkoa, Spain.
[3] Present address: Department of Physical Chemistry, Fritz-Haber-Institute of the Max-Planck-Society, 14195 Berlin, Germany.
[4] Donostia International Physics Center (DIPC), Paseo Manuel de Lardizabal 4, 20018 Donostia-San Sebastián Gipuzkoa, Spain.
[5] Ikerbasque, Basque Foundation for Science, María Díaz de Haro 3, 48009 Bilbao, Spain.
[6] Department of Electricity and Electronics, FCT-ZTF, University of the Basque Country (UPV/EHU), 48940 Leioa, Spain.
[†] Authors contributed equally.
[*] Correspondence to N.S.M. (niclasmueller@gmx.de), J.A. (aizpurua@ehu.eus), and J.J.B. (jjb12@cam.ac.uk).

Supplementary Experimental Data **2**
S1. Electronic Raman Scattering Background 2
S2. Analytical Models of Vibrational Pumping and Laser Heating 4
S3. Continuous-wave SERS 8
S4. Mixed SAM Characterisation 10

Theory and Simulation **11**
S5. Detailed Description of Simulations 11
S6. Characterisation of the Radiative Plasmonic Modes 13
S7. DFT Calculations of the Raman Tensors 14
S8. Simulations with Different Molecular Packing Densities 18
S9. Contributions of the Collective Vibrational Modes to the Raman Signal 20
S10. Collective Effects under Pulsed Illumination 21

References **25**

Supplementary Experimental Data

S1. Electronic Raman Scattering Background

Surface-enhanced Raman scattering experiments often show a strong background due to electronic Raman scattering (ERS) of electrons in the metal. In the pulsed SERS experiments presented in the main text, this ERS background depends on the intensity of the excitation laser and exhibits strongly non-linear scaling, especially in the anti-Stokes spectrum (see main text Fig. 2). Here, we analyse this background signal in more detail to understand excitations of the conduction electrons in the metal, which follow different physics than the molecular vibrations investigated in the main text.

Figure S1a shows the power-dependent SERS spectra presented in the main text (Fig. 2a) across a wider range of Raman shifts. This figure highlights that the spectra contain two components to the ERS background: (i) a contribution nearly constant across the entire spectrum and scaling super-linearly with laser power, and (ii) a contribution at low energies decaying rapidly to higher energies. We attribute these two contributions to different populations of electrons, the thermalised distribution of electrons in equilibrium with the metal lattice and a population of 'hot' carriers induced by the laser pulses[1]. Since thermalisation of hot electrons occurs on timescales <100 fs via electron-electron scattering[2], the use of pulsed lasers drastically enhances the importance of the latter population for the spectral background.

Recent experimental work[3] has shown that the ERS intensity of the thermalised electrons can be described with a Bose-Einstein distribution ($n_{BE}(\tilde{\nu},T) = [\exp(hc\tilde{\nu}/k_B T) - 1]^{-1}$), since the light inelastically scatters of (bosonic) waves of the electron gas[4]. In contrast, the population of hot electrons follows a Fermi-Dirac distribution ($n_{FD}(\tilde{\nu},T) = [\exp(hc\tilde{\nu}/k_B T) + 1]^{-1}$) since individual electrons are excited by the laser photons. The total anti-Stokes ERS signal S_{ERS} at Raman shift $\tilde{\nu}$ can hence be modelled with

$$S_{ERS}(\tilde{\nu}) \propto a_{lat} n_{BE}(-\tilde{\nu}, T_{lat}) + a_{hot} n_{FD}(-\tilde{\nu}, T_{hot}) \quad , \qquad \text{(S1)}$$

where a_{lat} and a_{hot} are the spectral amplitudes of thermalised and hot electrons, respectively, and account for the relative contribution of the two electronic populations. While the thermalised electron population is in equilibrium with the metal lattice at T_{lat}, the hot electron distribution should exhibit a much higher temperature T_{hot}. We fix T_{hot} corresponding to the energy of photons of the laser at $\hbar\omega_l = k_B T_l$, as it is impossible to extract the temperature of this flat background from the spectral range encompassed. With a 785 nm laser ($\hbar\omega_l$ = 1.6 eV), this corresponds to temperatures $T_l \approx$ 18,000 K giving a nearly flat background across the range of energies observable in the spectra (~3000 cm^{-1}). We note that representing this energy as an extreme temperature is misleading since the distribution is not thermalised and instead merely represents the pumping energy.

In Figure S1a,b the model for the ERS in equation (S1) is fit to the anti-Stokes background with free parameters a_{lat}, a_{hot} and T_{lat}. At high Raman shifts, the flat signal from hot electrons dominates (Figure S1a) while the contribution from thermalised carriers fits the background feature at low shifts well (Figure S1b).

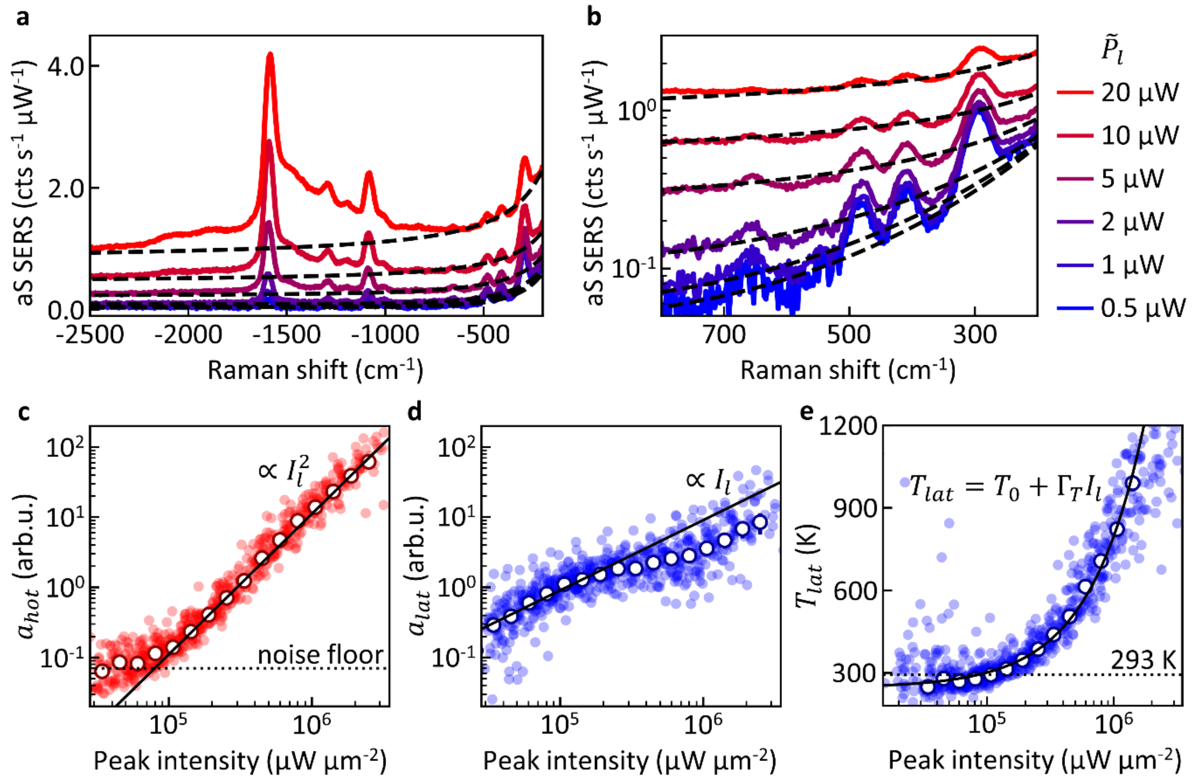

Figure S1: The electronic Raman scattering (ERS) background. **a,** Average anti-Stokes spectra of 70 NPoMs recorded with a pulsed laser (785 nm, 80 MHz, 0.5 ps) at different powers $\tilde{P}_l$ (colours). The ERS background is approximated with the model in equation (S1) (black dotted line). **b,** Power-dependent anti-Stokes spectra (colours) and ERS fit (black dotted line) at low Raman shifts. **c,** Spectral amplitude a_{hot} of the hot electron population from fitting spectra of individual particles. After overcoming the experimental noise, the signal scales quadratically with laser intensity. **d,** Spectral amplitude a_{lat} of the thermalised ERS contribution from the fit, scaling sub-linearly. **e,** Lattice temperature extracted from the thermalised ERS fit. A laser-induced heating model with $T_{lat} = T_0 + \Gamma_T I_l$ fits the data well. Filled points are data from 70 individual NPoMs, open circles are averages.

Fitting power-dependent spectra of 70 individual NPoM structures, the fit parameters give insight about the excitation of electronic states under pulsed laser illumination. As shown in Figure S1c, the population of hot electrons increases quadratically with coupling-corrected laser intensity (after overcoming the experimental detection limit). This observation is analogous to the quadratic scaling of the anti-Stokes signal in the vibrational pumping regime: a first photon excites the electron before a second photon is anti-Stokes scattered by the hot carrier (before relaxation can occur). We would term this thus an electron-pumping regime. In contrast, the thermalised ERS background shows sub-linear scaling (Figure S1d). This might be due to a reduction of the population of thermalised electrons as they are excited by the laser pulses. It also might be due to the time it takes to equilibrate the heated thermalised electrons, which can exceed the pulse duration so it is probed before heating has fully saturated.

Finally, we analyse the temperature extracted from the thermalised ERS background. We observe that the temperature rises linearly with laser intensity fitting a simple model of laser-induced heating $T_{lat} = T_0 + \Gamma_T I_l$. From this data, we estimate the heating rate $\Gamma_T \approx 0.5$ mK µW^{-1} µm^2. We note room temperature is slightly underestimated by this fit, due to slight corrections from the outcoupled spectrum modified by the plasmon resonance.

S2. Analytical Models of Vibrational Pumping and Laser Heating

We describe in this section a simple analytical model to characterise the presence of vibrational pumping in experiments. With this aim, we assume that the vibrational dynamics is given by a single collective vibrational mode consisting of an effective number N_{eff} of identical molecules, with all the molecules coupling with equal strength to a single plasmonic mode. This collective mode vibrates at frequency ω_{ϕ} and with decay rate γ_{ϕ}. The vibrational population n_{ϕ} of the mode can be separated into two different contributions: thermal population n_{ϕ}^{th} and population induced by vibrational pumping n_{ϕ}^{vp}. The thermal population is given by a Bose-Einstein distribution at temperature T according to $n_{\phi}^{th} = \left[\exp\left(\hbar\omega_{\phi}/k_b T\right) - 1\right]^{-1}$. Within the vibrational pumping regime ($n_{\phi}^{vp} \ll 1$), the population induced by vibrational pumping scales linearly with the laser intensity. Thus, introducing the vibrational pumping rate Γ_{vp} yields

$$n_{\phi} = n_{\phi}^{th} + n_{\phi}^{vp} = n_{\phi}^{th} + \Gamma_{vp}\, I_l \quad . \tag{S2}$$

A simple model can be used to relate the vibrational pumping rate Γ_{vp} to the properties of the plasmonic NPoM and molecules[5]. Within the vibrational pumping regime, we can obtain

$$\Gamma_{vp} = \frac{N_{eff}\sigma_{\mathrm{S}}}{\gamma_{\phi}\eta(\omega_{\mathrm{S}})\hbar\omega_{\mathrm{S}}} = \frac{N_{eff}}{\gamma_{\phi}\hbar\omega_{\mathrm{S}}}\frac{8\pi}{3}\left(\frac{Q_k^0 R_k}{4\pi\varepsilon_0 c_0}\right)^2 \omega_{\mathrm{S}}^4 L_m EF(\omega_l)^2\,\frac{\Gamma^T(\omega_S)}{\Gamma^0(\omega_S)} \quad , \tag{S3}$$

where we consider that the population induced by vibrational pumping scales linearly with the effective number of molecules N_{eff} [6] and where $\eta(\omega_{\mathrm{S}})$ is the radiative yield at the Stokes frequency ω_{S}. Additionally, in Eq. (S3) we have considered that the surface-enhanced Stokes scattering cross-section σ_S determining the amount of vibrational pumping is given by[5]

$$\sigma_{\mathrm{S}} = \frac{8\pi}{3}\left(\frac{Q_k^0 R_k}{4\pi\varepsilon_0 c_0^2}\right)^2 \omega_{\mathrm{S}}^4 L_m EF(\omega_l)^2\,\frac{\Gamma^R(\omega_S)}{\Gamma^0(\omega_S)} \ , \tag{S4}$$

with $Q_k^0 = \sqrt{\hbar/2\omega_{\phi}}$ the zero-point amplitude of the vibration, R_k the Raman tensor, ε_0 the vacuum permittivity, c_0 the speed of light in vacuum, $L_m = \left(\varepsilon_g + 2\right)/3$ the electromagnetic local field correction[7] (with gap permittivity ε_g = 2.1 in our system), $EF(\omega_l)$ the electromagnetic field enhancement factor at the laser frequency ω_l and $\Gamma^T(\omega_S)$, $\Gamma^R(\omega_S)$ and $\Gamma^0(\omega_S)$ the total, the radiative and the spontaneous (in a homogeneous media with ε_g = 2.1) decay rates at the Stokes frequency ω_S. Therefore, Γ_{vp} can be extracted either from the knowledge of the different parameters in Eq. S3, or experimentally from fitting the linear dependence of the population on the laser intensity (Eq. S2). Moreover, we define the threshold laser intensity I_l^{th} for vibrational pumping as the intensity at which the thermal and induced population are equal ($n_{\phi}^{th} = n_{\phi}^{vp}$), which yields

$$I_l^{th} = \frac{n_{\phi}^{th}}{\Gamma_{vp}} . \tag{S5}$$

The experimental fitting of I_l^{th} provides a more reliable measure for the vibrational pumping than Γ_{vp} since the absolute calibration of the phonon population or of the intensity emitted does not influence the result.

We plot in Figure S2a the dependence on the intensity of the experimental effective population [Eq. (9) in the main text] of six vibrational modes averaged over 70 NPoMs (open circles), and the linear fitting obtained numerically (solid lines) assuming no heating (n_{ϕ}^{th}constant at T = 293 K). The values of Γ_{vp} obtained from the fitting are plotted in Figure S2b (black circles and error bar). We have verified that the values of Γ_{vp} of the high wavenumber vibrational modes are approximately proportional to the peak amplitudes in the Stokes spectrum in Fig. 2b of the main text, which is consistent with the occurrence of vibrational pumping. However, a more accurate analysis of this relationship would need to take into account the different collective enhancement experienced by each of the vibrational modes and other phenomena such as Intramolecular Vibrational Redistribution (IVR). Using Eq. S5 we also extract the experimental values of I_l^{th} (Figure S2c). These values assume that the increase on population is due to vibrational pumping and not to heating of the sample (i.e. an increase of n_{ϕ}^{th}), which is not guaranteed particularly for low energy vibrations.

In the following, we examine the validity of the assumption of no heating with the simple model presented in this section. For this purpose, we calculate the effective number of molecules that corresponds to the experimentally fitted values of Γ_{vp}. We focus on the three vibrational modes discussed in the main text (ω_{ν} = 292 cm^{-1}, 1080 cm^{-1}, and 1585 cm^{-1}) and use approximate values of $EF(\omega_l)^2$ and $P_F(\omega_S)$ from the electromagnetic simulations (Supplementary Information Sections S5 and S6). More specifically, we use $EF(\omega_l)^2$ = 50^2, as well as $\Gamma^T(\omega_S)/\Gamma^0(\omega_S)$ = 285^2, 340^2, and 337^2 for the modes at 292 cm^{-1}, 1080 cm^{-1}, and 1585 cm^{-1}, respectively. Additionally, we use $\hbar\gamma_{\phi}$ = 5 meV (see Supplementary Information Sections S5) and the Raman tensors R_k in Table S1.

For the high wavenumber modes 1080 cm^{-1} and 1585 cm^{-1}, the vibrational pumping rate Γ_{vp} obtained from the fit of the experimental results corresponds approximately to $N_{eff} \approx$ 400, which is within an order of magnitude of the experimental value. This difference is reasonable considering the assumptions of the simple model in this section, which is only intended to convey the main qualitative features in a more intuitive manner than the full model used in the main text. Notably, Fig. 4f of the main text indicates that the assumed linear scaling of vibrational pumping with N_{eff} fails for large N_{eff}. More importantly, within the simple model in this section the low wavenumber mode at 292 cm^{-1} requires a significantly larger effective number of molecules, $N_{eff} \approx$ 1200. N_{eff} can vary slightly for different vibrational modes (if more than one plasmonic mode is considered) but the much larger value obtained for the 292 cm^{-1} mode suggests the existence of an additional mechanism exciting the low wavenumber vibrational modes beyond the optomechanically-induced vibrational pumping, such as laser-induced heating and intramolecular vibrational redistribution. We emphasize that the same conclusion can be reached using the much more rigorous model used in the main text (which includes the effect of many collective modes, the full plasmonic response of the NPoM cavity and the exact coupling of each molecule to the NPoM cavity). Specifically, when using this improved model, we could not account for the experimental increase of the S_{aS}/S_S ratio for the low-energy vibrational mode at 292 cm^{-1} without including heating.

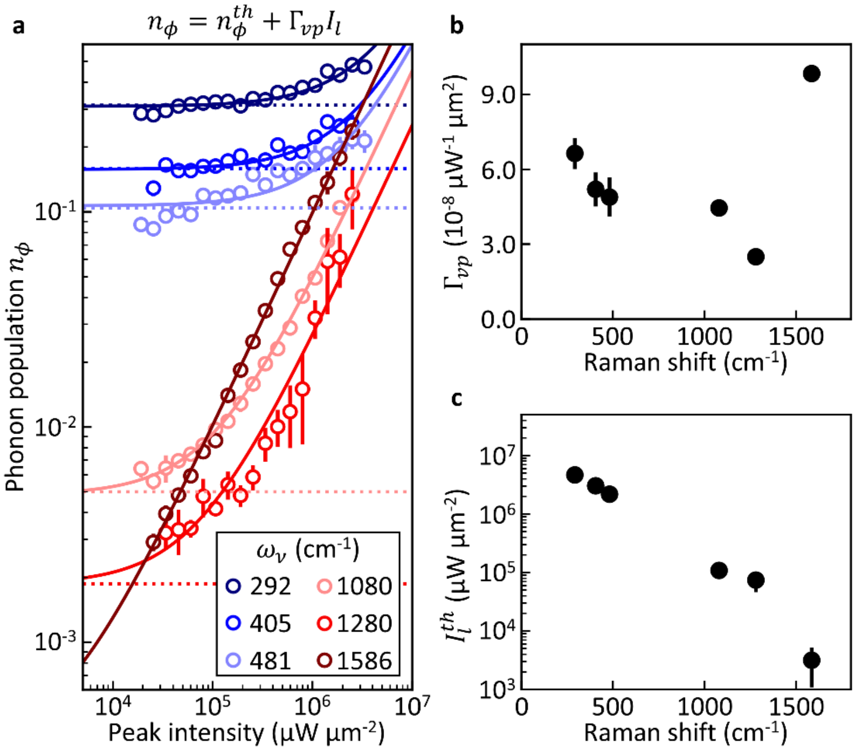

Figure S2: Analytical model of vibrational pumping. **a,** Vibrational populations extracted from the anti-Stokes to Stokes ratio for six vibrational modes. Open circles indicate averages of 70 NPoMs, with error bars showing standard errors. Populations are corrected to match thermal population at room temperature for low laser intensities. Dotted lines are thermal populations at 293 K, solid lines fits with analytical model $n_\phi = n_\phi^{th} + \Gamma_{vp} I_l$. The analytical model fits the data well. **b,** Vibrational pumping rates Γ_{vp} extracted from the analytical model. **c,** Laser intensity threshold for vibrational pumping extracted from the analytical model.

Finally, we consider the effect of laser heating to show that it may be behind the behaviour of the low wavenumber vibrational modes, but that its effect should be negligible for the high wavenumber ones. In Figure S3, we fit the effective vibrational population of the same six vibrational modes as before but now using a simple laser heating model[8] which assumes that the effective vibrational population is entirely thermalised ($n_\phi = n_\phi^{th}$), with an intensity-dependent temperature increase T = 293 K + $\Gamma_T I_l$ (and thus an intensity dependent n_ϕ^{th}). Here, Γ_T is the rate of laser-induced heating. We first fit independently the population of each vibrational mode (Figure S3a), using Γ_T as a free parameter. The fitting obtained for the lower wavenumber modes is quite satisfactory but becomes much worse for the highest wavenumber modes. Furthermore, very different values for Γ_T are obtained for the different modes, corresponding to very different temperatures (at I_l = 10^6 µW µm^{-2}, we find T = 324 K, 515 K, and 1457 K for the vibrational modes at 292 cm^{-1}, 1080 cm^{-1}, and 1585 cm^{-1}, respectively).

Critically, these results cannot be the consequence of a fully thermalized system, as this would require a single value Γ_T for all the vibrational modes. Next, we fix the value $\Gamma_T \approx$ 0.04 mK/(µW µm^{-2}) obtained from the fitting of the 292 cm^{-1} vibrational mode. We chose this value because it is obtained from the lowest energy vibrational mode, and thus it determines the maximum heating possible (larger values of Γ_T would lead to larger vibrational populations of the 292 cm^{-1} mode, assuming no additional unknown mechanism decreasing the vibrational population below the thermal one; notice that we are not in the optomechanical cooling regime). We then plot the populations that this value of Γ_T provides for the rest of vibrational modes (Figure S3b) and no vibrational pumping. The obtained results are in relatively good agreement with the effective population of the low wavenumber modes

extracted experimentally. In contrast, the populations of the high wavenumber vibrational modes calculated in this way strongly disagree with the corresponding experimental effective populations. Therefore, this analysis indicates that laser-induced thermal heating may be behind the increase of the population of the low wavenumber vibrational modes, but that the population of the high wavenumber vibrational modes is dominated by vibrational pumping in this system, with only a small contribution from laser heating.

Last, we remark that the fitting of the vibrational populations with this laser-heating model provides a very different value of Γ_T than that obtained from fitting the ERS background in Section S1. Since the NPoM system is driven outside thermal equilibrium by pulsed laser excitation here, apparent temperatures experienced by electrons in the metal and vibrations of molecules can vary. Hence, different heating rates of the two sub-systems are expected due to the different mechanisms of excitation with different efficiencies.

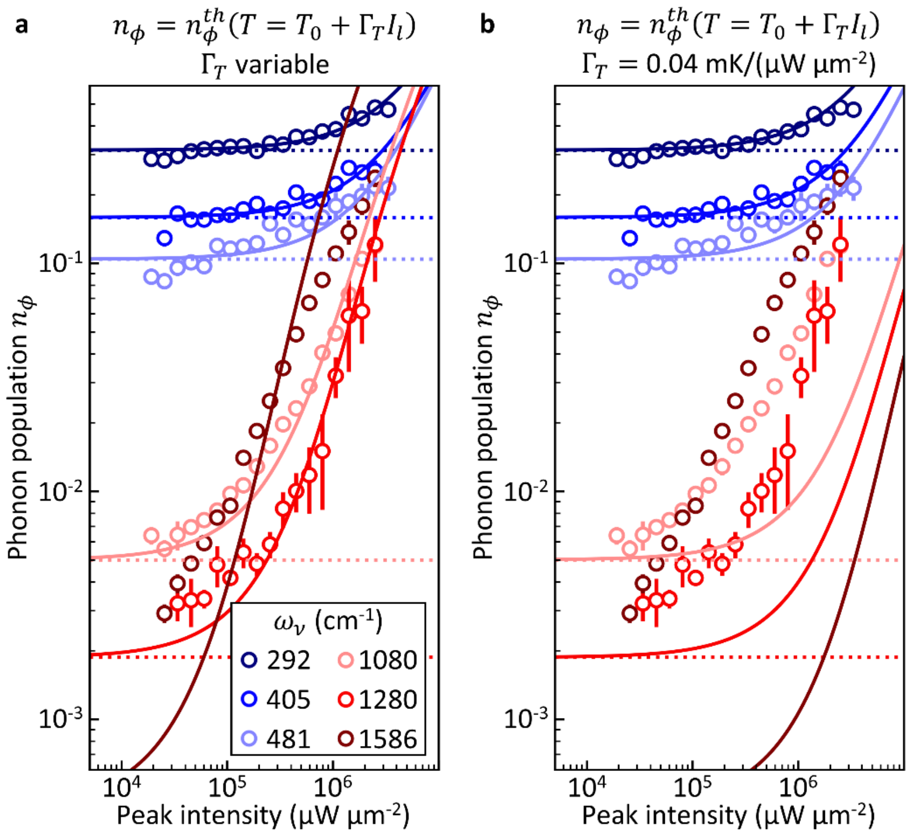

Figure S3: Analytical model of laser-induced heating. **a,b** Vibrational populations extracted from the anti-Stokes to Stokes ratio for six vibrational modes. Open circles indicate averages of 70 NPoMs, with error bars showing standard errors. Populations are corrected to match thermal population at room temperature for low laser intensities. Dotted lines are thermal populations at 293 K, solid lines are fits with a laser-heating model $n_\phi = n_\phi^{th}(T)$ with $T = 293$ K $+ \Gamma_T I_l$. In (a), the rate of laser-induced heating rate Γ_T is varied independently for all modes, while it is fixed at Γ_T = 0.04 mK/(μW μm^{-2}) in (b).

S3. Continuous-wave SERS

Using a continuous-wave (CW) laser at 785 nm wavelength, we investigate whether vibrational pumping is also observed without a pulsed laser. Analogous to the experiments described in the main text, power-dependent SERS experiments are carried out with the CW laser. Sweeping the average CW laser power from 5 µW to 200 µW, SERS spectra averaged over many NPoMs scale linearly with laser power (see Figure S4), in contrast to strongly non-linear scaling of the pulsed SERS spectra in main text Fig. 2.

Extracting the SERS signal of the same vibrational modes as in main text Fig. 3, both Stokes and anti-Stokes signals for all modes exhibit linear scaling (see Figure S5). Hence, the S_{aS}/S_S ratio is constant over the entire range of coupling-corrected laser intensities probed in the experiment (10^1 to 10^3 µW µm^{-2}). Similar to pulsed experiments, the vibrational population at room temperature is underestimated due to different field enhancement of Stokes and anti-Stokes emission. Only the 1586 cm^{-1} mode shows a slight super-linear increase at highest laser intensities indicating the possible onset of vibrational pumping between 10^3 and 10^4 µW µm^{-2} for NPoM cavities with particularly large enhancement, consistent with pulsed experiments. For the 1080 cm^{-1} vibration, we obtain a vibrational pumping threshold of 10^5 µW µm^{-2} under pulsed illumination (see main text Fig. 3e). Exciting the NPoM with a CW laser of such high intensity would strongly damage the nanostructure and molecules. Therefore, the high peak intensities of the pulsed laser are required for this investigation of molecular optomechanics in NPoMs at room temperature. Vibrational pumping is likely observed with CW lasers only at cryogenic temperatures.[9]

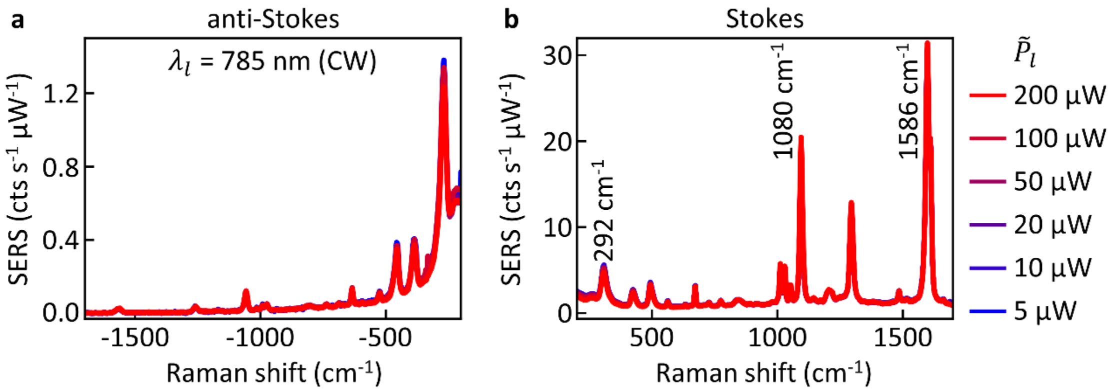

Figure S4: Power-dependent CW SERS spectra. a, anti-Stokes and **b,** Stokes SERS spectra of BPT in NPoM nanocavities, excited with a CW laser (785 nm) with average power $\tilde{P}_l$ ranging from 5 to 200 µW (colours). Spectra were sorted by coupling-corrected laser power, averaged over 80 NPoMs, and normalised by integration time and excitation power.

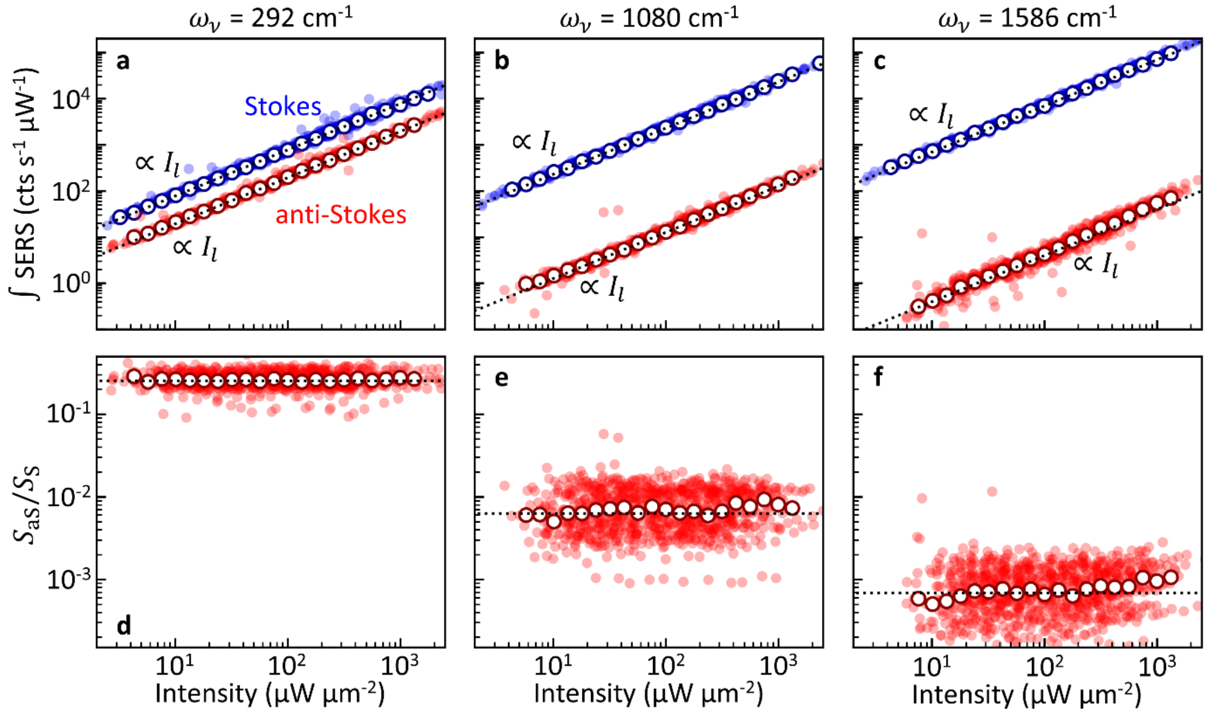

Figure S5: SERS signal *vs* CW laser intensity. a-c, Extracted Stokes and anti-Stokes signal *vs* coupling-corrected laser intensity for (a) 292 cm^{-1}, (b) 1080 cm^{-1} and (c) 1586 cm^{-1} vibrational modes. **d-f,** Anti-Stokes to Stokes ratio for the (d) 292 cm^{-1}, (e) 1080 cm^{-1} and (f) 1586 cm^{-1} vibrational modes. Filled points are data from 80 individual NPoMs, open circles are averages, dotted lines show the indicated scaling law.

S4. Mixed SAM Characterisation

To characterize the mixed self-assembled monolayers (SAMs) of the molecules BPT and QTH, we conducted CW SERS experiments (Figure S6). Average SERS spectra, recorded on ~100 NPoMs for each mixing fraction, are shown in main text Figure 4b and Figure S6a. The spectra are dominated by the SERS bands of BPT, while a much weaker peak at 1320 cm^{-1} indicates the presence of QTH. To assess the mixing, we first calculate histograms of the peak area ratio $A_{QTH}/(A_{QTH}+A_{BPT})$, see Figure S6b. We find a narrow distribution of peak ratios, which confirms a homogeneous mixing of the two molecules on the length scale that is probed by each NPoM hotspot. Because of the very weak QTH SERS intensity, we use the decrease in BPT SERS intensity to quantitatively measure the dilution of the SAM (Figure S6c). The wide distribution of SERS signal on each sample is due to varying coupling efficiencies of individual NPoMs. Hence, a large sample size is necessary to estimate dilutions. The relative decrease of A_{BPT} in Figure S6c is larger than the relative increase of A_{QTH} in Figure S6b, which likely occurs due to changes of the SERS cross section of the QTH molecules in the mixed SAMs. Such changes were not observed for BPT in previous experiments with several types of mixed SAMs, where SERS intensities were compared to XPS signals[10]. We therefore use the BPT SERS intensity in Figure S6 to quantitatively measure the dilution.

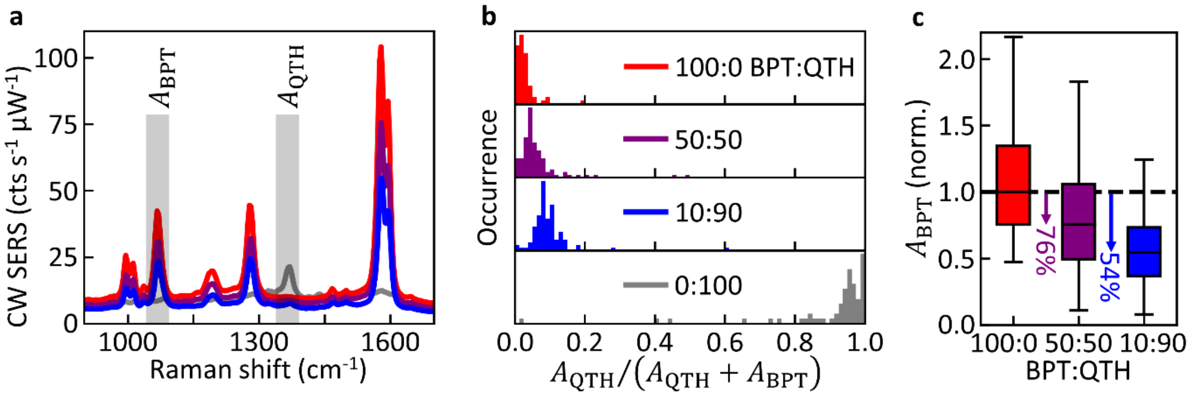

Figure S6: Characterisation of mixed SAMs. a, Average CW Raman spectra from ~100 NPoMs for SAMs with different ratio of BPT to QTH mixing in solution (see labels in b). The areas of the indicated SERS peaks of BPT, A_{BPT}, and QTH, A_{QTH}, (grey shaded) are analysed in b,c. **b,** Histograms of ratios of QTH SERS signal to sum of QTH and BPT signal from ~100 NPoMs for each sample with different mixing fraction in solution. Narrow histograms on each sample indicate good mixing of BPT and QTH without the formation of domains. **c,** Boxplots of BPT SERS signal on each sample, normalised to the median of the 100% BPT sample. The actual dilution of BPT on the sample can be estimated from the reduction in BPT SERS signal.

Theory and Simulation

S5. Detailed Description of Simulations

The theoretical model used to describe the optomechanical interaction between the NPoM cavity and the BPT molecules and, thus, to obtain the vibrational population and the Raman signal can be found in Ref. 11. This model is based on a continuum-field formalism within cavity quantum electrodynamics that incorporates the full plasmonic response of the NPoM cavity via the electromagnetic Green's function[12,13]. Additionally, the model allows for including many molecules inside the cavity and incorporates the molecule-molecule interactions mediated by the plasmonic structure (direct dipole-dipole interactions are neglected). These interactions and the dissipation processes are included in the master equation that governs the vibrational dynamics and that needs to be solved to obtain the desired response. The model neglects the influence of intramolecular vibrational redistribution and other effects on the thresholds of laser intensity required to reach the vibrational pumping regime. We describe in this section the system considered in our calculations (molecules, NPoM cavity and illumination), focusing on the properties that determine the values of the input parameters of the quantum master equation.

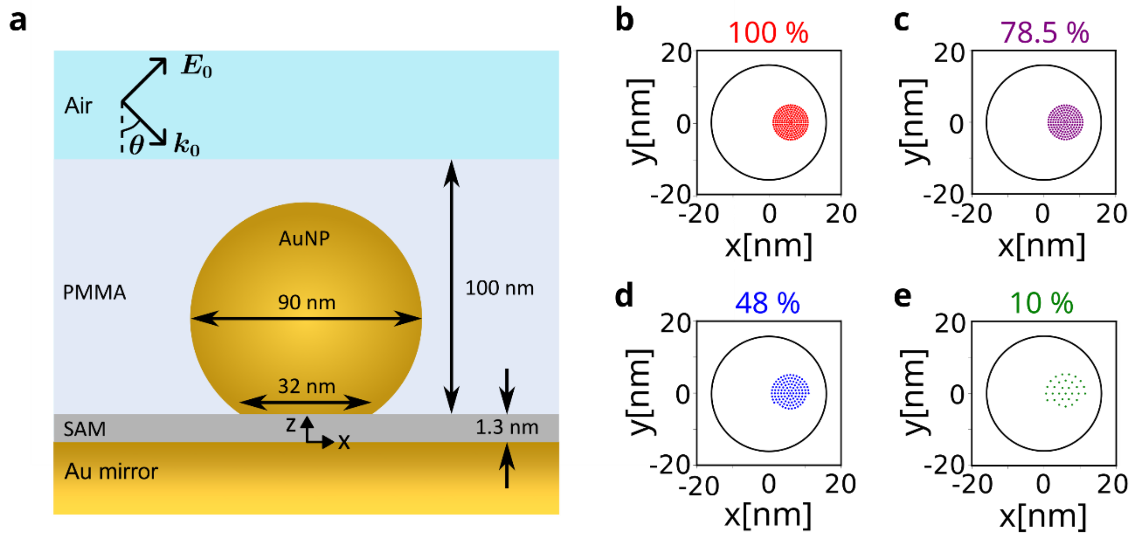

Figure S7: Sketch of the simulated NPoM cavity and the different molecular patches used to obtain the Raman response for different BPT packing densities. a, An Au spherical nanoparticle (AuNP) and an Au mirror are separated by a 1.3 nm thick dielectric layer with dielectric constant ε_g = 2.1. The nanoparticle has a 90 nm diameter and is truncated at the bottom, forming a circular flat facet of 32 nm diameter. The AuNP is covered by a 100 nm thick PMMA layer with refractive index n = 1.49. The system is illuminated with a p-polarized plane-wave with field amplitude E_0, wavenumber k_0 and oblique incidence (θ = 55°). The sketch is not to scale. **b-e,** Molecular patches used to simulate different experimental BPT molecular packing densities, namely: (b) 100%, (c) 78.5 %, (d) 48%, and (e) 10%. Dots are used to represent the position of the molecules in the patch. The molecular patches are located on the *xy*-plane at z = 0.65 nm. In these patches the area is fixed at ≈ 72 nm^2, while the number of molecules changes from 217 to 30 (see Table S3).

The NPoM cavity is composed by an Au spherical nanoparticle of 90 nm diameter, which is placed at nanometric distance over a gold substrate and truncated at the bottom by a circular flat facet of 32 nm diameter (see sketch in Figure S7a). The gold permittivity of both the spherical nanoparticle and the substrate is taken from experiments in Ref. 14. The gap between the Au substrate and the Au spherical nanoparticle is 1.3 nm thick and has permittivity ε_g = 2.1 (corresponding to the estimated permittivity of the self-assembled monolayer (SAM) of BPT molecules treated as a homogeneous layer). We also introduce a 100 nm thick layer of permittivity ε_{PMMA} = 1.49 covering the Au spherical nanoparticle to simulate the additional PMMA layer in the experiments[15]. The external illumination consists of a p-polarized plane-wave with oblique incidence (θ = 55°, see Figure S7a), field amplitude E_0 and 785 nm wavelength. The origin of the coordinates system is set at the interface between the Au substrate and the BPT SAM (see Figure S7a), with the *z*-axis normal to that interface and aligned with the centre of the spherical nanoparticle. Additionally, in the calculation of the propagator matrices $K^{\pm}$ [Eq. (6) of the main text], we consider that the detector is in the backward direction $\mathbf{r}_d = [-\sin(\theta)\mathbf{x} + \cos(\theta)\mathbf{z}] \cdot 1000 \text{ nm}$.

We consider that the vibrational decay rate of the BPT molecules is $\hbar\gamma_\nu$ = 5 meV. This decay rate is larger than the intrinsic vibrational decay rate of the BPT molecules to effectively include the broadening induced by the pulsed illumination in experiments. Additionally, the Raman tensors are obtained from DFT calculations of a BPT molecule that interacts with two flat Au surfaces[3]. These surfaces represent the bottom of the nanoparticle and top of the substrate. The new values of the Raman tensors of the BPT molecules are summarized in Table S1 (see Section S7 for further information on the DFT simulations).

Furthermore, the molecules are treated as point-like dipoles and placed on the horizontal *xy*-plane at half the distance between the flat facet of the spherical nanoparticle and the Au substrate (i.e. at *z* = 0.65 nm in Figure S7a). In general, the number of molecules N_{total} required to fill the entire NPoM gap is beyond our current computational capabilities. Therefore, we perform simulations with up to N_{sim} = 217 molecules placed within a circular patch (see the patches in Figure S7b-e). The centre of the patch is not located at the centre of the gap, but it is displaced 6 nm distance in the *x*-direction (except in the configuration of Figure S10e) towards a position where the local field-enhancement is generally large, as shown in Section S6. The molecules are placed along concentric rings around a molecule at the centre of the circular patch. The distance d between adjacent rings is fixed and we place $6n$ molecules in the n-th ring, so that the distance between adjacent molecules in all rings is $\approx d$. More exactly, this distance is equal to $nd\sqrt{2 - 2\cos(\pi/3n)}$, which takes values from d (for the first ring) to $d\pi/3$ (for large enough n values). The exact position of the patch and of each molecule is depicted in Figure S7 and Figure S10.

Table S1: Raman tensors of the BPT molecules. R_{ij} is in units of $\varepsilon_0\text{Å}^2\text{amu}^{-1/2}$. ω_ν is the frequency of the molecular vibrational mode.

ω_ν [cm^{-1}]	R_{xx}	$R_{xy} = R_{yx}$	$R_{xz} = R_{zx}$	R_{yy}	$R_{yz} = R_{zy}$	R_{zz}
292	20.5	4.2	51.4	4.1	9.2	179.1
1080	- 40.8	- 7.6	- 130.7	- 7.3	- 28.0	- 484.0
1585	- 53.1	- 26.5	- 162.8	- 13.4	- 94.0	- 728.6

S6. Characterisation of the Radiative Plasmonic Modes

The electromagnetic response of the NPoM cavity under plane-wave illumination is calculated using the Radio Frequency Module of COMSOL Multiphysics[16]. In these simulations, the geometry of the universe considered is spherical and perfectly matched layers are included to facilitate the convergence of the numerical results. The scattering cross-section is shown in Figure 1d in the main text and exhibits two main peaks at wavelengths 660 nm and 882.5 nm. Additionally, we show in Figure S8a the spectra of the near field-enhancement calculated at different positions of the molecular patch in Figure S7b, which consists of 217 molecules separated by ≈ 0.6 nm (Section S5)[17]. The positions at which we plot the enhancement are marked by coloured dots in the inset of Figure S8a, with each dot corresponding to a single molecule. We focus on the absolute value of the near-field enhancement in the z-direction $|E_z|/E_0$ (with E_0 the amplitude of the incident electric field), because (i) the plasmonic field is strongly polarized in this direction, and (ii) the Raman dipoles of the molecular vibrations are also mostly oriented in this direction.

The near-field enhancement spectra show four peaks at ≈ 660 nm, ≈ 690 nm, ≈ 882.5 nm and ≈ 977.5 nm, which indicates that four plasmonic modes are excited. We plot in Figure S8b-e the spatial distribution of the plasmonic field enhancement $|E_z|/E_0$ in the horizontal *xy*-plane at the centre of the NPoM gap obtained at these four resonant frequencies. The circumferences marked with dashed lines in Figure S8b-e indicate the size and position of the molecular patch in the simulations (except for the simulations in Fig. S10), showing that the patch centre is approximately located at the position where the local-field is maximum for the plasmonic resonances at 660 nm, 690 nm and 977.5 nm. Two of the plasmonic resonances exhibit mirror symmetry with respect to the *y*-axis (corresponding to the peaks at $\lambda \approx$ 660 nm and 882.5 nm, see Figure S8a), whereas the other two exhibit rotational symmetry (peaks at $\lambda \approx$ 690 nm and 977.5 nm). These four plasmonic resonances are similar to those found in previous works[18,19]. We remark that the field distribution at each resonance frequency is not perfectly symmetric because more than one mode is simultaneously excited. Finally, as the laser wavelength is 785 nm, the anti-Stokes photons associated with the 1080 cm^{-1} and 1585 cm^{-1} vibrational modes are emitted at $\lambda \approx$ 723 nm and 698 nm, respectively, close to the plasmonic resonance at ≈ 690 nm (Figure S8c). On the other hand, the Stokes photons from the 1080 cm^{-1} and 1585 cm^{-1} vibrational modes are emitted at $\lambda \approx$ 858 nm and 897 nm, respectively, near the plasmonic resonance at ≈ 882.5 nm (Figure S8d).

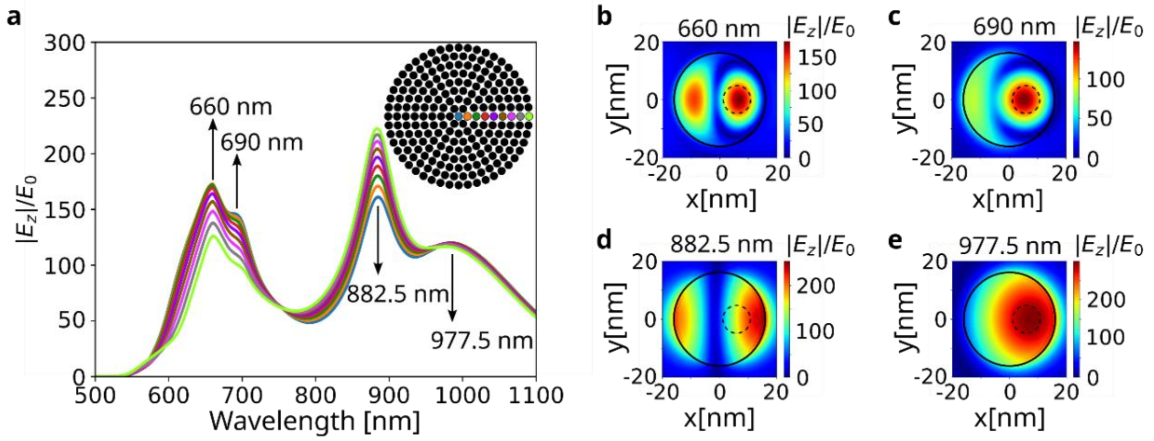

Figure S8: Near-field enhancement spectra and spatial distribution of the plasmonic modes. a, Dependence of the near-field enhancement $|E_z|/|E_0|$ on the laser wavelength at the different positions of the molecular patch that are indicated in the inset (same patch as in Figure S7b). The patch is composed of 217 molecules and located at half the distance between the Au nanoparticle facet and the Au substrate. The position associated with each spectrum is marked by the colour according to the inset, where the molecules are represented by dots. **b-e,** Spatial distribution of the near-field enhancement $|E_z|/|E_0|$ on the *xy*-plane at the centre of the gap (*z* = 0.65 nm), for excitation at the wavelength of the four plasmonic resonances at (b) 660 nm, (c) 690 nm, (d) 882.5 nm, and (e) 977.5 nm. The circumferences marked with solid lines represent the size of the circular facet at the bottom of the spherical nanoparticle, whereas those marked with dashed lines indicate the size and position of the molecular patch (corresponding to the area covered by the inset in (a)).

S7. DFT Calculations of the Raman Tensors

We describe in this section the details of the calculation of the Raman tensors of the BPT molecules attached to two gold monolayer planes of 15 and 16 atoms. This calculation has been discussed in detail in Ref. 3 and we summarize it here for completeness. We first compute the relaxed structure of the BPT molecule bound to the gold planes (the molecule interacts covalently with the gold monolayer plane formed by 15 atoms), which corresponds to a situation of local energy minimum (see Figure S9). We then obtain the vibrational frequencies and the Raman tensors of the vibrational modes for this relaxed structure.

We carry out the calculation of the relaxed molecular structure of BPT sandwiched between the gold planes at DFT level in two steps. We first perform periodic boundary conditions calculations to simulate the structure of a cavity that consists of a self-assembled monolayer (SAM) of BPT molecules between two gold slabs, where each slab consists of 8 layers compatible with a unit cell (2x3) of the Au (111) surface. We locate 2 BPT molecules in the unit cell to simulate the SAM. We use the code VASP (version 5.4.4)[20–22] to optimize the structure of the cavity. To describe the non-covalent interactions between the molecules in the SAM, and between the molecules and the gold slabs, we select the exchange-correlation functional OPTPBE[23]. We sample the reciprocal space using a Monkhorst-Pack mesh formed by 2x3 points. We use a basis set that consists of a series of plane waves with a cutoff energy of 420 eV and the PAW pseudopotentials extracted from the VASP database[24,25]. To ensure that the optimization of the structure of the SAM in the cavity is properly converged, we impose a convergence criterion for the electronic energy of 10^{-6} eV, and of 0.01 eV/Å for the forces acting along all the degrees of freedom of the atoms of the molecules and along the direction perpendicular to the slabs for the gold atoms that delimit the cavity. These periodic boundary

conditions calculations set the position of the gold atoms (two Au(111) monolayer planes of fifteen and sixteen atoms that delimit the cavity) for the next step. In this second step, we carry out an additional DFT calculation to relax the structure of a single BPT molecule interacting with the two selected Au planes. We fix a distance between the Au planes of 1 nm and freeze the positions of the atoms within the Au planes to avoid the collapse of the cavity. We use the exchange-correlation functional B3LYP[26] and, to account properly for the non-covalent interactions between the molecule and the Au planes, we use the empirical correction D3[27] with the damping function proposed by Becke and Johnson[28,29]. We select the basis set LANL2DZ for the atoms of gold, and the Gaussian basis set 6-31G(d,p) for the atoms of hydrogen, carbon and sulphur. We carry out this calculation with the code Gaussian16 Rev B.01.[30]

Last, we perform a third DFT calculation to obtain the vibrational frequencies and the Raman tensors of the vibrational modes for the optimised structure (a single BPT molecule located between two gold planes) obtained in the previous step. To isolate the native vibrational modes of the BPT molecule from the vibrational modes delocalized over the gold planes and the molecule, we freeze the degrees of freedom of the gold atoms during the calculation of the vibrational frequencies and the Raman tensors. We use the code Gaussian16 Rev B.01 and the same DFT functional and basis sets as for the second DFT optimisation. We show in Table S1 the Raman tensor obtained in this way for three selected vibrational modes. We show in Table S2 the positions (in Cartesian coordinates) of the atoms that compose the structure of a single BPT molecule sandwiched between two Au planes, which we use to obtain the Raman tensors.

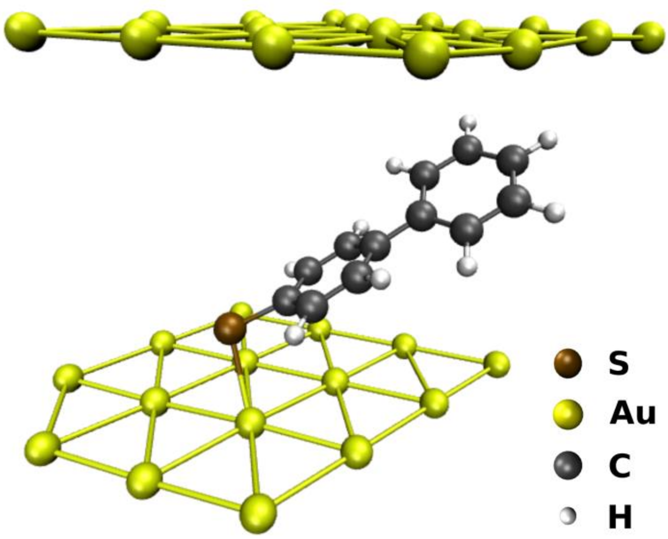

Figure S9: Atomistic model of the structure of the BPT molecule attached to two gold monolayer planes. This structure, which corresponds to a situation of local energy minimum, is used to compute the vibrational frequencies and Raman tensors for the selected vibrational modes. The spheres coloured in brown, yellow, grey and white represent the atoms of sulphur, gold, carbon and hydrogen, respectively.

Table S2: Cartesian coordinates of the structure of a single BPT molecule interacting with two Au planes. Column 1: Chemical symbols of the atoms that compose the structure of the BPT molecule and the Au planes. **Columns 2-4:** X, Y and Z coordinates in Å of the positions of the atoms shown in column 1.

Atomic symbol	X	Y	Z
H	10.5464390458	3.3834109849	9.3595297027
H	8.7291153085	2.4618070615	7.9719122982
H	6.9907558025	6.3865313469	7.6392980548
H	8.8536041254	7.3233095493	8.9465818338
H	9.7290626349	7.5979961191	10.920681886
H	11.5888170822	8.6523686787	12.132282474
H	14.2377959356	5.5386907174	10.8011393425
H	12.4042088495	4.5344470708	9.4991433278
C	9.7841262387	4.0496636694	8.9713215526
C	8.7560131357	3.5207529423	8.203781260
C	7.7229851829	4.3585596906	7.7416533831
C	7.7693990383	5.7365532970	8.0216803308
C	8.8147339535	6.2545702889	8.7703319139
C	9.8347084440	5.4241301942	9.2784719411
C	10.9261016872	5.9939235813	10.0962613067
C	10.7174139735	7.1550044090	10.8647103495
C	11.7645631301	7.7433764955	11.5688882722
C	13.0390021683	7.1683568060	11.5485260951
C	13.2512002547	5.9907687961	10.8263635739
C	12.2094294478	5.4153693840	10.1015422231
S	6.3034041295	3.6580969428	6.9625625842
H	13.8558660053	7.6436293001	12.0817304829
Au	11.5881119783	1.8485190710	14.6805489521
Au	2.7141170000	1.8485680000	14.6817440000
Au	5.6720870000	1.8485680000	14.6686240000

Au	8.6300574322	1.8485665424	14.6658065154
Au	13.0667768931	4.4101035986	14.6446640975
Au	4.1930970000	4.4102480000	14.6897940000
Au	7.1510670000	4.4102480000	14.6620040000
Au	10.1090388917	4.4103208355	14.7488401099
Au	14.5459336705	6.9719710186	14.7703422367
Au	5.6720870000	6.9719280000	14.6594540000
Au	8.6300564386	6.9719270637	14.6883388157
Au	11.5880072084	6.9719163320	14.6553212504
Au	16.0249770000	9.5335980000	14.6651040000
Au	7.1510667537	9.5335975977	14.6624332359
Au	10.109036563	9.5335980756	14.6836538435
Au	13.067003689	9.5335970606	14.6958579277
Au	2.9579621569	0.0000036361	4.8738559442
Au	5.9159318595	0.0000026072	4.8158951390
Au	8.8739037707	-0.0000035338	4.8462928482
Au	1.4789801023	2.5616899488	4.8748924873
Au	4.4370048530	2.5615780122	4.8200981219
Au	7.3949963839	2.5617355067	4.9219548890
Au	10.3528899647	2.5616645605	4.8555129396
Au	2.9579700607	5.1233302174	4.8704250753
Au	5.9160715273	5.1235319990	4.8490965268
Au	8.8739193552	5.1233385961	4.8628854332
Au	11.8318799209	5.1233503462	4.8772311223
Au	4.4369294381	7.6850185502	4.8818407048
Au	7.3949204073	7.6850306709	4.8747122894
Au	10.3528906305	7.6850298602	4.8746712203
Au	13.3108600506	7.6850298265	4.8726732732

S8. Simulations with Different Molecular Packing Densities

In this section we provide further details of the simulations of the Raman signal in Figure 4 of the main text, where the dependence on the packing density of the BPT molecules is studied. Experimentally, the packing density of the BPT molecules is manipulated by mixing them with QTH molecules in different proportions, because the SERS spectrum of the latter molecules does not overlap with the vibrational lines of interest of the BPT molecules.

In the simulations, only BPT molecules are considered and we control the packing density by changing the intermolecular distances d (see Section S5 for details on the definition of d and on how the molecules are distributed into concentric rings to keep the distance between neighbouring molecules relatively constant). To set d, we first consider the total number of BPT molecules filling the entire NPoM gap of 32 nm diameter. For distances d between adjacent rings of the molecular patch equal to 0.6 nm, 0.68 nm, 0.85 nm and 1.8 nm we estimate the presence of $\approx$ 2100, $\approx$ 1650, $\approx$ 1000, and $\approx$ 200 molecules in the gap, respectively. Therefore, considering that d = 0.6 nm corresponds to the case of 100% BPT packing density, d = 0.68 nm, 0.85 nm and 1.8 nm correspond to the presence of $\approx$ 78.5%, $\approx$ 48% and $\approx$ 10% of molecules, respectively. These data are summarized in the first three columns of Table S3.

However, simulating the vibrational response of thousands of molecules is beyond our current computational capabilities. Thus, once the value of d has been determined for each BPT packing density, we simulate molecular patches of smaller size. In the case of d = 0.6 nm, the molecular patch consists of 217 molecules and is depicted in Figure S7b. The area covered by this patch is equal to $\approx$ 72 nm^2. We use this patch in the simulations in Figure 3 of the main text. Further, we also consider in Figure 4 in the main text the other values of the intermolecular distance for patches of similar area $\approx$ 72 nm^2 and thus of different number N_{sim} of molecules in the patch. These changes thus mimic the change of BPT packing density in the experiments. In particular, to mimic the experiments where $\approx$ 78.5%, $\approx$ 48% and $\approx$ 10% of the molecules are present, we use molecular patches consisting of 169, 113 and 30 molecules (corresponding to d = 0.68 nm, 0.85 nm and 1.8 nm), respectively. We depict these patches in Figure S7c-e, and the corresponding N_{sim} and area are summarized in the last two columns of Table S3.

For completeness, we also perform simulations where the number of BPT molecules in the patch is fixed at 217, whereas the effective area of the patch is modified so that d remains the same as in Fig. 4 of the main text (d = 0.68 nm, 0.85 nm and 1.8 nm). The resulting anti-Stokes to Stokes ratios are plotted in Figure S10a and show similar tendencies than simulations in main text Fig. 4, but with slightly worse matching of experimental results. The corresponding molecular patches are depicted in Figure S10b-e.

Table S3: Data of the molecular patches of fixed area for the different BPT molecular packing densities. % BPT denotes the BPT molecular packing density, d the distance between the different rings of the circular patches, N_{total} the total estimated number of molecules filling the whole NPoM cavity for this packing density, N_{sim} the number of molecules in the patches plotted in Figure S7b-e and used in the Raman calculations in Figure 3 (100% BPT) and Figure 4 of the main text, and A the area filled by the N_{sim} molecules.

**% BPT**	d **[nm]**	N_{total}	N_{sim}	A **[nm²]**
100	0.6	≈ 2100	217	72
≈ 78.5	0.68	≈ 1650	169	71
≈ 48	0.85	≈ 1000	113	72
≈ 10	1.80	≈ 200	30	72

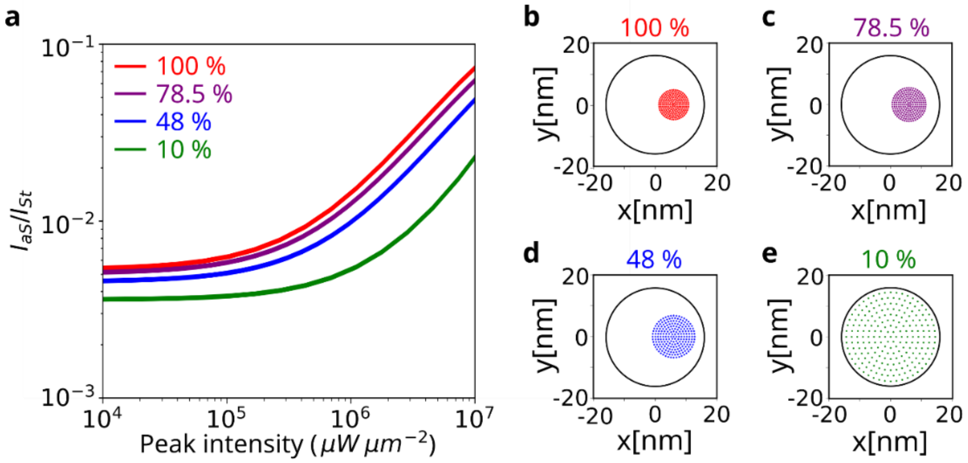

Figure S10: SERS signal simulated for different BPT molecular packing densities using patches with fixed number of molecules. a, Anti-Stokes to Stokes ratio as a function of the laser intensity for the different intermolecular distances that mimic the different BPT packing densities, namely: 100 % (d = 0.6 nm, red line), 78.5 % (d = 0.68 nm, purple line), 48 % (d = 0.85 nm, blue line), and 10 % (d = 1.8 nm, green line). All patches contain 217 molecules, so that the area of the patch increases with decreasing packing density. **b-e,** Sketch of the molecular patches used for the simulations in (a). The circumference marked with a solid black line correspond to the flat facet of the Au nanoparticle.

S9. Contributions of the Collective Vibrational Modes to the Raman Signal

We have presented in Figure 5 of the main text the populations of the collective modes (indexed by the integer ϕ) for the 1080 cm^{-1} and 1586 cm^{-1} vibrational modes of the BPT molecules and a molecular patch consisting of 217 molecules at molecular distances d = 0.6 nm (see Figure S7b for a sketch of the patch). We have shown that the population of the collective mode ϕ = 1 captures well the effective population from the anti-Stokes to Stokes ratio in the case of the 1080 cm^{-1} vibrational mode (Figure 5a of the main text), whereas this effective population has important contributions from the collective modes ϕ = 1 and ϕ = 2 in the case of the 1586 cm^{-1} mode (Figure 5b of the main text). Here, we complement this discussion by analysing the linewidths, optomechanical spring shifts (i.e., the frequency shifts induced by the plasmonic response of the NPoM cavity to the collective vibrational modes) and contributions to the anti-Stokes spectrum of the different collective modes, which can be obtained from Eqs. (4) and (5) in the main text.

With this aim, we rewrite Eqs. (4) and (5) in the main text as $dP^{St}/d\Omega = \sum_{\phi=1}^{N}(dP^{St}/d\Omega)_{\phi}$ and $dP^{aS}/d\Omega = \sum_{\phi=1}^{N}(dP^{aS}/d\Omega)_{\phi}$, where

$$\left(\frac{dP^{St}}{d\Omega}\right)_{\phi} = Re\frac{[S^{-1}(1+B)K^{+}S]_{\phi\phi}}{i(\omega-\omega_l+\lambda_{\phi})} \quad , \tag{S6}$$

$$\left(\frac{dP^{aS}}{d\Omega}\right)_{\phi} = Re\frac{[S^{-1}B^{T}K^{-}S]_{\phi\phi}}{i(\omega-\omega_l-\lambda_{\phi}^{*})} \quad , \tag{S7}$$

correspond to the contribution of the collective mode ϕ to the Stokes and anti-Stokes differential scattered power, respectively. Additionally, Eqs. (S6) and (S7) indicate that the optomechanical spring shift and linewidth of the collective mode ϕ are given by $\mathrm{Re}\,\lambda_{\phi}$ and $2\left|\mathrm{Im}\,\lambda_{\phi}\right|$, respectively.

We first show in Figure S11a-b the linewidths and optomechanical spring shift of all collective modes at fixed laser intensity I_l = 1.2·10^{7} µW/µm^{2}. For the 1080 cm^{-1} mode, the collective mode ϕ = 1 (blue dot in Figure S11a) is characterized by the largest linewidth and optomechanical spring shift. On the other hand, in the case of the 1586 cm^{-1} mode, the collective mode ϕ = 1 is again the one with largest linewidth (blue dot in Figure S11b), but mode ϕ = 2 is the one with largest optomechanical spring shift (orange dot in Figure S11b).

Furthermore, we plot in Figure S11c-d the contributions to the anti-Stokes spectrum of the collective modes ϕ = 1 (solid blue line), ϕ = 2 (orange line) and ϕ = 3 (green line), at the same value of laser intensity. We note that some of these contributions are negative, however the total anti-Stokes spectrum (dashed black line) is always positive. In these figures, the *x*-axis corresponds to the frequency detuning of ω with respect to the anti-Stokes frequency in the thermal regime, $\omega_{las}+\omega_{\nu}$. For the 1080 cm^{-1} mode, the total anti-Stokes spectrum can be well approximated by the contribution from the collective mode ϕ = 1 only, and the vibrational response can be well understood by only considering this single collective mode. However, in the case of the 1586 cm^{-1} mode, both collective modes ϕ = 1 and ϕ = 2 contribute significantly to the anti-Stokes spectrum in Figure S11d (dashed black line), and the latter is well reproduced by the sum of these two contributions (dotted red line). This result is consistent with the behaviour of the effective population of this vibrational mode in the main text (see Figure 5b), which is not well reproduced neither by the population of the collective mode ϕ = 1 nor by the population of ϕ = 2.

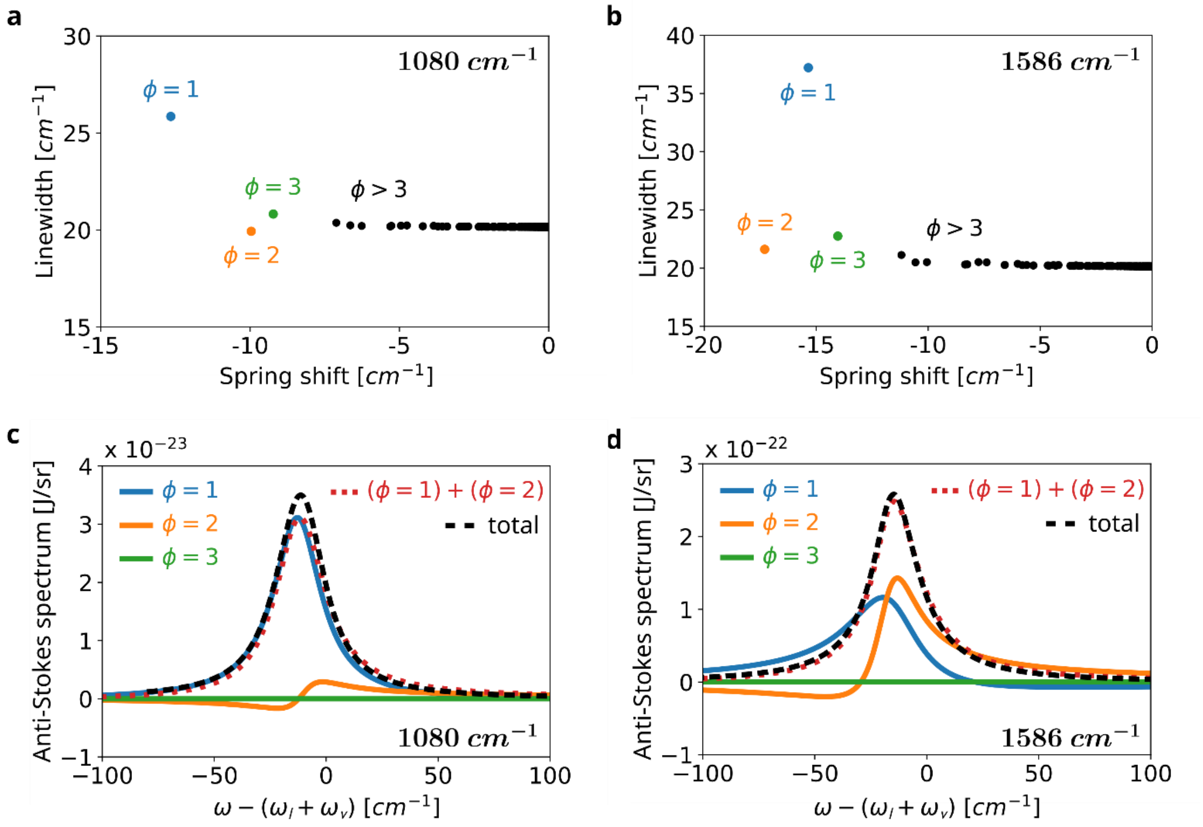

Figure S11: Contributions of the different collective modes to the vibrational response. a,b, Linewidths and optomechanical spring shifts of the different collective vibrational modes indexed by ϕ for the vibrations at (a) 1080 cm^{-1} and (b) 1586 cm^{-1}. A molecular patch consisting of 217 molecules at molecular distances d = 0.6 nm is considered (see Figure S7b). **c,d,** Contributions of the collective vibrational modes ϕ = 1 (solid blue line), ϕ = 2 (solid orange line), ϕ = 3 (solid green line) to the total anti-Stokes spectrum (dashed black line) for the vibrations at (c) 1080 cm^{-1} and (d) 1586 cm^{-1} and the same molecular patch as in (a)-(b), as a function of the detuning of ω with respect to the anti-Stokes frequency $\omega_{las} + \omega_v$ in the thermal regime. Dotted red line represents the sum of the contributions of the collective vibrational modes ϕ = 1 and ϕ = 2. The laser intensity is fixed at I_l = 1.2·10^7 µW/µm^2.

S10. Collective Effects under Pulsed Illumination.

The theoretical calculations provided in the main text, as well as in the previous sections of this Supplementary Information, consider CW illumination with effectively broadened vibrational decay rates. In this section, we show that this optomechanical model can be used to describe the experiments conducted with pulsed illumination. To this end, we perform simulations of the Raman signal scattered from a few molecules under pulsed illumination and under CW illumination and show that they agree very well. To obtain this agreement, it is crucial to increase the vibrational decay rate in the CW simulations, mimicking the effective broadening induced in the Raman peaks by the pulsed illumination. Further, we show that vibrational collective effects also emerge in the Raman signal under pulsed illumination, which is consistent with the results provided in the main text.

To facilitate the simulation of the Raman signal under pulsed illumination, we consider in this section a single plasmonic mode and N identical molecules. The optomechanical Hamiltonian is thus given by[5,6,31]

$$\hat{H}(t) = \hbar\omega_{pl}\hat{a}^\dagger\hat{a} + \sum_{j=1}^{N}\left[\hbar\omega_v\hat{b}_j^\dagger\hat{b}_j - \hbar g_0\hat{a}^\dagger\hat{a}(\hat{b}_j^\dagger + \hat{b}_j) + \hbar\Omega(t)(\hat{a}^\dagger e^{-it\omega_l} + \hat{a}e^{it\omega_l})\right], \quad \text{(S8)}$$

where $\hat{a}^\dagger$ and $\hat{a}$ are the creation and annihilation plasmonic operators, $\hat{b}_j^\dagger$ and $\hat{b}_j$ are the creation and annihilation vibrational operators of molecule j, ω_{pl} is the resonance frequency of the plasmonic mode, g_0 is the optomechanical coupling rate (considered equal for all the molecules), ω_l is the laser frequency, and $\Omega(t)$ is the pumping rate of the plasmonic mode. $\Omega(t)$ is time-dependent in the case of pulsed-illumination and time-independent in the case of CW illumination [$\Omega(t) = \Omega_0$, see below]. In both cases, the pumping rate is proportional to the square root of the laser intensity. The dynamics of the full system is described by the master equation

$$\frac{\partial}{\partial t}\hat{\rho} = -\frac{i}{\hbar}\left[\hat{H}(t), \hat{\rho}\right] + \frac{\kappa}{2}\mathcal{D}[\hat{a}]\hat{\rho} + \sum_{j=1}^{N}\left(\frac{\gamma_v^{in}(1 + n_v^{th})}{2}\mathcal{D}\left[\hat{b}_j\right]\hat{\rho} + \frac{\gamma_v^{in}n_v^{th}}{2}\mathcal{D}\left[\hat{b}_j^\dagger\right]\hat{\rho}\right), \quad \text{(S9)}$$

where $\hat{\rho}$ is the density matrix of the full system, κ the decay rate of the plasmonic mode, γ_v^{in} the intrinsic vibrational decay rate, n_v^{th} the thermal vibrational population, and $\mathcal{D}\left[\hat{O}\right]\hat{\rho} = 2\hat{O}\hat{\rho}\hat{O}^\dagger - \hat{O}^\dagger\hat{O}\hat{\rho} - \hat{\rho}\hat{O}^\dagger\hat{O}$ the standard Lindblad dissipator, with $\hat{O}$ an arbitrary operator. n_v^{th} and γ_v^{in} are identical for all molecules.

The calculations are further simplified by transforming expressions into the rotating frame at the laser frequency and introducing the fluctuation operators $\delta\hat{a} = \hat{a} - \alpha(t)$ and $\delta\hat{b}_j = \hat{b}_j - \beta(t)$, where

$$\alpha(t) = \frac{\Omega(t)}{\kappa/2 + i\Delta_{pl}}, \qquad \beta(t) = \frac{g_0|\alpha(t)|^2}{\omega_v - i\gamma_v^{in}/2} \quad \text{(S10)}$$

are the coherent amplitudes of the plasmonic and vibrational modes, respectively, at time t [taking into account that $\kappa \gg 4g_0\mathrm{Re}(\beta)$ in our system] [31]. Here, $\Delta_{pl} = \omega_{pl} - \omega_l$ is the detuning of the laser with respect to the plasmonic resonance frequency. After this transformation by replacing $\delta\hat{a} \rightarrow \delta\hat{a}\, e^{i\,\arg[\alpha(t)]}$ [with arg[α(t)] the argument of α(t), see Ref. 5], and linearizing the Hamiltonian, we obtain

$$\hat{H}_{lin}(t) = \hbar\Delta_{pl}\delta\hat{a}^\dagger\delta\hat{a} + \sum_{j=1}^{N}\left[\hbar\omega_v\delta\hat{b}_j^\dagger\delta\hat{b}_j - \hbar g_0|\alpha(t)|\left(\delta\hat{a}^\dagger + \delta\hat{a}\right)\left(\delta\hat{b}_j^\dagger + \delta\hat{b}_j\right)\right]. \quad \text{(S11)}$$

The dynamics of the full system is governed by the master equation

$$\frac{\partial}{\partial t}\hat{\rho} = -\frac{i}{\hbar}\left[\hat{H}_{lin}(t), \hat{\rho}\right] + \frac{\kappa}{2}\mathcal{D}[\delta\hat{a}]\hat{\rho} + \sum_{j=1}^{N}\left(\frac{\gamma_v^{in}(1 + n_v^{th})}{2}\mathcal{D}\left[\delta\hat{b}_j\right]\hat{\rho} + \frac{\gamma_v^{in}n_v^{th}}{2}\mathcal{D}\left[\delta\hat{b}_j^\dagger\right]\hat{\rho}\right). \quad \text{(S12)}$$

We emphasize that the Hamiltonian $\hat{H}_{lin}(t)$ [Eq. (S11)] is now time-dependent under pulsed illumination, because $\alpha(t)$ depends on $\Omega(t)$ [Eq. (S10)], making numerical calculations more elaborate for a large number of molecules. In our simulations, we consider that the pulsed laser intensity has a gaussian shape given by

$$I(t) = I_0\exp\left[-\left(\frac{t2\sqrt{\ln 2}}{\tau_p}\right)^2\right], \quad \text{(S13)}$$

where τ_p is the duration of the pulse [defined as the full width at half maximum (FWHM) of the pulse] and I_0 the peak intensity. Thus, the pumping rate $\Omega(t)$ under pulsed illumination is given by

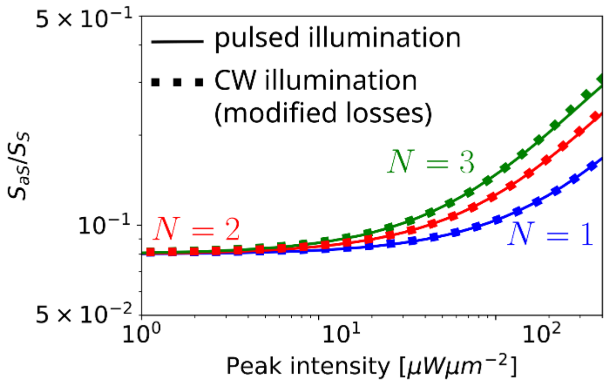

Figure S122: Comparison of the simulated anti-Stokes to Stokes ratio under CW illumination (with modified vibrational losses) and pulsed illumination. We plot the dependence of the simulated anti-Stokes to Stokes ratios on the peak intensity I_0 under pulsed illumination (solid lines) and CW illumination (with modified vibrational losses, dotted lines). The results are plotted for increasing number of molecules: $N = 1$ (blue), $N = 2$ (red), and $N = 3$ (green). The simulations under pulsed illumination are obtained using Eqs. (S12), (S14) and (S16), and considering $\tau_p = 0.5$ ps and intrinsic vibrational losses $\hbar\gamma_v^{in} = 1.5$ meV. The simulations under CW illumination are performed using Eqs. (S12) and (S17), with $\Omega(t) = \Omega_0$ and by increasing the value of the vibrational decay rate to 5 meV, which mimics the effective broadening that the pulses induce in the Raman peaks in the experiments. We fix the following values in all the simulations $\hbar\omega_v = 134$ meV, $\hbar\omega_{pl} = 1.797$ eV, $\hbar g_0 = 32$ meV, and $\hbar\kappa = 136$ meV.

$$\Omega(t) = \Omega_0 \exp\left[-\frac{1}{2}\left(\frac{t2\sqrt{\ln 2}}{\tau_p}\right)^2\right], \tag{S14}$$

where Ω_0 can be estimated from the peak intensity I_0 through[5,31]

$$\Omega_0 = I_0 \sqrt{\frac{3\pi c^2 \kappa\eta}{2\hbar\omega_{pl}^3}}, \tag{S15}$$

with η the radiative yield. In contrast, $\hat{H}_{lin}$ [Eq. (S11)] is time-independent under CW illumination. In this case, $\Omega(t) = \Omega_0$ can be related again to the laser intensity I_0 through Eq. (S15).

We show next that the simulations of the anti-Stokes to Stokes ratio under CW illumination (with modified vibrational losses) and pulsed illumination give almost identical results. To this end, we consider the 1080 cm^{-1} vibrational mode of the BPT molecule ($\hbar\omega_v = 134$ meV) and a plasmonic mode at 690 nm ($\hbar\omega_{pl} = 1.797$ eV). Additionally, we fix $\hbar g_0 = 32$ meV and $\hbar\kappa = 136$ meV, following Ref. 12 (which obtained these values from numerical fittings of the optical response of the NPoM cavity). The radiative yield is $\eta = 0.25$. Further, in the simulations with pulsed illumination, we consider $\tau_p = 0.5$ ps (corresponding to the duration of the pulses in our experiments) and $\hbar\gamma_v^{in} = 1.5$ meV (estimated from experimental spectra). We numerically solve the master equation in Eq. (S12), with the time-dependent pumping rate in Eq. (S14), and compute the integrated Raman spectrum [32,33]

$$S_{pulse}(\omega) \propto \int_0^\infty dt \int_{-\infty}^\infty d\tau e^{-i\omega\tau} \langle \delta\hat{a}^\dagger(t+\tau)\delta\hat{a}(t)\rangle, \tag{S16}$$

where the expected value is evaluated for an initial room temperature thermal state, given by the product of thermal plasmonic and vibrational density matrices.

On the other hand, in the simulations with CW illumination, we solve again the Master equation in Eq. (S12), but considering $\Omega(t) = \Omega_0$. Crucially, to mimic the behaviour of the Raman signal found under pulsed illumination (where the Raman peaks are broadened due to the pulse duration[31]), we modify the value of the vibrational decay rate used in the CW simulations. We estimate the value of this effective broadening from the FWHM of experimental spectra and find $\hbar\gamma_v^{in} \rightarrow \hbar\gamma_v = 5$ meV. We then compute the CW Raman spectrum (with this modification of the vibrational decay rate) as

$$S_{CW}(\omega) \propto \int_{-\infty}^{\infty} d\tau e^{-i\omega\tau} \langle \delta\hat{a}^{\dagger}(\tau)\delta\hat{a}(0)\rangle_{ss}, \tag{S17}$$

where the expected value is evaluated at the steady state (ss). The factor ω^4 that is sometimes included in the calculation of the SERS spectra to account for the frequency dependence of the dipolar emission is ignored in Eqs. (S16) and (S17), for simplicity.

We plot in Fig. S12 the anti-Stokes to Stokes ratios S_{aS}/S_S obtained under pulsed illumination (solid lines) and under CW illumination (with modified vibrational losses, dotted lines), as a function of I_0 and for increasing number of molecules $N = 1$ (blue), $N = 2$ (red), and $N = 3$ (green). We find an excellent agreement between the simulations obtained under pulsed illumination and under CW illumination (with suitable modification of the vibrational decay rate). Crucially, both simulations find an almost identical modification of the intensity threshold required to reach the vibrational pumping regime with increasing N. This threshold intensity scales with $1/N$, in agreement with the scaling shown in the main text at small values of N (Fig. 4f). Therefore, we conclude that CW simulations can be used to analyse the collective optomechanical response of the system also under pulsed illumination, at least for the pulse durations considered in our experiments.

References

1. Wu, S. *et al.* The connection between plasmon decay dynamics and the surface enhanced Raman spectroscopy background: Inelastic scattering from non-thermal and hot carriers. *J. Appl. Phys.* **129**, 173103 (2021).
2. Saavedra, J. R. M., Asenjo-Garcia, A. & García De Abajo, F. J. Hot-Electron Dynamics and Thermalization in Small Metallic Nanoparticles. *ACS Photonics* **3**, 1637–1646 (2016).
3. Boehmke Amoruso, A. *et al.* Uncovering low-frequency vibrations in surface-enhanced Raman of organic molecules. *Nat. Commun.* **15**, 6733 (2024).
4. Devereaux, T. P. & Hackl, R. Inelastic light scattering from correlated electrons. *Rev. Mod. Phys.* **79**, 175–233 (2007).
5. Schmidt, M. K., Esteban, R., Benz, F., Baumberg, J. J. & Aizpurua, J. Linking classical and molecular optomechanics descriptions of SERS. *Faraday Discuss.* **205**, 31–65 (2017).
6. Zhang, Y., Aizpurua, J. & Esteban, R. Optomechanical Collective Effects in Surface-Enhanced Raman Scattering from Many Molecules. *ACS Photonics* **7**, 1676–1688 (2020).
7. Le Ru, E. C. & Etchegoin, P. G. *Principles of Surface-Enhanced Raman Scattering*. (Elsevier, 2009).
8. Tschannen, C. D. *et al.* Tip-Enhanced Stokes-Anti-Stokes Scattering from Carbyne. *Nano Lett.* **22**, 3260–3265 (2022).
9. Maher, R. C., Etchegoin, P. G., Le Ru, E. C. & Cohen, L. F. A Conclusive Demonstration of Vibrational Pumping under Surface Enhanced Raman Scattering Conditions. *J. Phys. Chem. B* **110**, 11757–11760 (2006).
10. Mueller, N. S. *et al.* Collective Mid-Infrared Vibrations in Surface-Enhanced Raman Scattering. *Nano Lett.* **22**, 7254–7260 (2022).
11. Jakob, L. A. *et al.* Giant optomechanical spring effect in plasmonic nano- and picocavities probed by surface-enhanced Raman scattering. *Nat. Commun.* **14**, 3291 (2023).

12. Zhang, Y. *et al.* Addressing Molecular Optomechanical Effects in Nanocavity-Enhanced Raman Scattering beyond the Single Plasmonic Mode. *Nanoscale* **13**, 1938–1954 (2021).

13. Dezfouli, M. K. & Hughes, S. Quantum optics model of Surface Enhanced Raman Spectroscopy for arbitrarily shaped plasmonic resonators. *ACS Photonics* **4**, 1245–1256 (2017).

14. Johnson, P. B. & Christy, R. W. Optical Constants of the Noble Metals. *Phys. Rev. B* **6**, 4370–4379 (1972).

15. Beadie, G., Brindza, M., Flynn, R. A., Rosenberg, A. & Shirk, J. S. Refractive index measurements of poly(methyl methacrylate) (PMMA) from 0.4–1.6 µm. *Appl. Opt.* **54**, F139–F143 (2015).

16. COMSOL Multiphysics v. 5.5. www.comsol.com. *COMSOL AB, Stockholm, Sweden*.

17. Matei, D. G., Muzik, H., Gölzhäuser, A. & Turchanin, A. Structural Investigation of 1,1'-Biphenyl-4-thiol Self-Assembled Monolayers on Au(111) by Scanning Tunneling Microscopy and Low-Energy Electron Diffraction. *Langmuir* **28**, 13905–13911 (2012).

18. Esteban, R. *et al.* The morphology of narrow gaps modifies the plasmonic response. *ACS Photonics* **2**, 295–305 (2015).

19. Tserkezis, C. *et al.* Hybridization of plasmonic antenna and cavity modes: Extreme optics of nanoparticle-on-mirror nanogaps. *Phys. Rev. A* **92**, 053811 (2015).

20. Kresse, G. & Hafner, J. Ab initio molecular dynamics for liquid metals. *Phys. Rev. B* **47**, 558 (1993).

21. Kresse, G. & Furthmüller, J. Efficiency of ab-initio total energy calculations for metals and semiconductors using a plane-wave basis set. *Comput. Mater. Sci.* **6**, 15 (1996).

22. Kresse, G. & Furthmüller, J. Efficient iterative schemes for ab initio total-energy calculations using a plane-wave basis set. *Phys. Rev. B* **54**, 11169 (1996).

23. Klimeš, J., Bowler, D. R. & Michaelides, A. Chemical accuracy for the van der Waals density functional. *J. Condens. Phys. Condens. Matter* **22**, 022201 (2010).

24. Kresse, G. & Hafner, J. Norm-conserving and ultrasoft pseudopotentials for first-row and transition elements. *J. Phys. Condens. Matter* **6**, 8245 (1994).

25. Kresse, G. & Joubert, D. From ultrasoft pseudopotentials to the projector augmented-wave method. *Phys. Rev. B* **59**, 1758 (1999).

26. Becke, A. D. A new mixing of Hartree–Fock and local density-functional theories. *J. Chem. Phys.* **98**, 1372 (1993).

27. Grimme, S., Antony, J., Ehrlich, S. & Krieg, H. A consistent and accurate ab initio parametrization of density functional dispersion correction (DFT-D) for the 94 elements H-Pu. *J. Chem. Phys.* **132**, 154104 (2010).

28. Grimme, S., Ehrlich, S. & Goerigk, L. Effect of the damping function in dispersion corrected density functional theory. *J. Comput. Chem.* **32**, 1456 (2011).

29. Becke, A. D. & Johnson, E. R. A density-functional model of the dispersion interaction. *J. Chem. Phys.* **123**, 154101 (2005).

30. Frisch, M. J. *et al.* Gaussian 16, Revision B.01. (2016).

31. Lombardi, A. *et al.* Pulsed Molecular Optomechanics in Plasmonic Nanocavities: From Nonlinear Vibrational Instabilities to Bond-Breaking. *Phys. Rev. X* **8**, 011016 (2018).

32. Laussy, F.P., del Valle, E. & Tejedor, C. Luminescence spectra of quantum dots in microcavities. I. Bosons. *Phys. Rev. B* **79**, 235325 (2009).

33. Yamaguchi, M., Lyasota, A. & Yuge, T. Theory of Fano effect in cavity quantum electrodynamics. *Phys. Rev. Research* **3**, 013037 (2021).
